# Sulfatase 2 Is Associated with Steroid Resistance in Childhood Nephrotic Syndrome

**DOI:** 10.3390/jcm10030523

**Published:** 2021-02-02

**Authors:** Shipra Agrawal, Richard F. Ransom, Saras Saraswathi, Esperanza Garcia-Gonzalo, Amy Webb, Juan L. Fernandez-Martinez, Milan Popovic, Adam J. Guess, Andrzej Kloczkowski, Rainer Benndorf, Wolfgang Sadee, William E. Smoyer, on behalf of the Pediatric Nephrology Research Consortium (PNRC)

**Affiliations:** 1 Center for Clinical and Translational Research, Abigail Wexner Research Institute at Nationwide Children’s Hospital, Columbus, OH 43205, USA; chief@funite.science (R.F.R.); grudvica@gmail.com (M.P.); Adam.Guess@nationwidechildrens.org (A.J.G.); rbenndo@gmx.net (R.B.); 2 Department of Pediatrics, The Ohio State University College of Medicine, Columbus, OH 43210, USA; Andrzej.Kloczkowski@nationwidechildrens.org; 3 Battelle Center for Mathematical Medicine at Abigail Wexner Research Institute at Nationwide Children’s Hospital, Columbus, OH 43205, USA; saraswathi55@gmail.com; 4 Department of Mathematics, University of Oviedo, 33033 Oviedo, Spain; espe@uniovi.es (E.G.-G.); jlfm@uniovi.es (J.L.F.-M.); 5 Department of Biomedical Informatics, The Ohio State University College of Medicine, Columbus, OH 43210, USA; Amy.Hite@osumc.edu; 6 Department of Cancer Biology and Genetics, Center for Pharmacogenomics, The Ohio State University College of Medicine, Columbus, OH 43210, USA; sadee.1@osu.edu

**Keywords:** steroid resistant nephrotic syndrome, sulfatase 2, vascular endothelial growth factor (VEGF), glucocorticoids, FSGS

## Abstract

Glucocorticoid (GC) resistance complicates the treatment of ~10–20% of children with nephrotic syndrome (NS), yet the molecular basis for resistance remains unclear. We used RNAseq analysis and in silico algorithm-based approaches on peripheral blood leukocytes from 12 children both at initial NS presentation and after ~7 weeks of GC therapy to identify a 12-gene panel able to differentiate steroid resistant NS (SRNS) from steroid-sensitive NS (SSNS). Among this panel, subsequent validation and analyses of one biologically relevant candidate, sulfatase 2 (SULF2), in up to a total of 66 children, revealed that both SULF2 leukocyte expression and plasma arylsulfatase activity Post/Pre therapy ratios were greater in SSNS vs. SRNS. However, neither plasma SULF2 endosulfatase activity (measured by VEGF binding activity) nor plasma VEGF levels, distinguished SSNS from SRNS, despite VEGF’s reported role as a downstream mediator of SULF2’s effects in glomeruli. Experimental studies of NS-related injury in both rat glomeruli and cultured podocytes also revealed decreased SULF2 expression, which were partially reversible by GC treatment of podocytes. These findings together suggest that SULF2 levels and activity are associated with GC resistance in NS, and that SULF2 may play a protective role in NS via the modulation of downstream mediators distinct from VEGF.

## 1. Introduction

Nephrotic syndrome (NS) is among the most common kidney diseases seen in both children and adults [1]. The primary therapy for NS, oral glucocorticoids (GC), fails to induce a remission in ~10–20% of children, which either present with or develop GC resistance later in their course [2,3]. The known anti-inflammatory and immunosuppressive effects of GC have long served as indirect evidence that their mechanism of action in NS involves the inhibition of soluble mediators released by T lymphocytes [4]. Moreover, the presence of circulating factors in the plasma of NS patients that mediate disease has also been speculated for several decades. However, research has also highlighted the importance of renal podocytes as the primary target of injury in NS, and several monogenic causes have been attributed to the genes expressed primarily in podocytes [1,5,6]. Overall, the majority of NS patients present with no known underlying etiology and the molecular basis for clinical resistance to GC therapy remains largely unclear [1,7]. Thus, improving our understanding of molecular factors that determine clinical GC resistance offers a highly desirable strategy to develop more effective and targeted future therapies for NS.

In recent years, unbiased RNASeq analysis has become an excellent approach for identifying the molecular bases of diseases with unknown etiologies. Moreover, in addition to the conventional statistical methods, advanced “machine learning systems” have recently been used to address the challenges of analyzing large data sets generated from systems biology approaches, such as transcriptomics. These approaches have been successful in identifying genes or biomarkers that are disease-causing, useful for prognosis, or predictive of drug efficacy and optimal therapies [8,9,10,11,12,13,14,15,16,17,18].

In the current study, we performed transcriptome-wide gene sequencing and analysis of circulating leukocytes from patients with steroid-sensitive NS (SSNS) and steroid resistant NS (SRNS) to better understand the molecular basis of SSNS vs. SRNS. The peripheral blood leukocytes were collected from children both at clinical presentation with NS and after ~7 weeks of GC therapy, recruited through the Pediatric Nephrology Research Consortium (PNRC). Using a discovery cohort, we identified a gene panel using in silico algorithm-based approaches that could distinguish SSNS from SRNS, Pre and Post therapy and one of the top distinguishing and relevant genes, SULF2, was then studied in greater detail in a validation cohort. SULF2 was particularly selected as it has been previously reported to play crucial roles in podocyte physiology and maintenance of glomerular integrity, both of which are critically affected in NS [19,20,21].

Sulfatases are enzymes that catalyze the formation of sulfate esters from a variety of substrates, including carbohydrates, proteins, steroids, glycosaminoglycans, etc. SULF2 (endoglucosamine-6-sulfatase 2) primarily releases sulfate from various disaccharide units (e.g., IdoA2S-GlcNS6S, UA2S-GlcNS6S, UA-GlcNS6S) of heparin/heparan sulfate chains, thus remodeling the cell’s surface and modulating transmembrane signaling processes [22,23,24,25,26]. For example, SULF2 and its paralog, SULF1, are thought to affect VEGF bioavailability in glomerular endothelial cells, which is likely to remodel the subendothelial space and fenestrations, as was suggested by both SULF1/2 and VEGF conditional knock-out mice [19,27,28]. Thus, after identifying SULF2 to be differentially associated with SSNS and SRNS, pre- and post-therapy, we performed subsequent analyses for its expression, activity, and downstream effects on VEGF bioavailability in both patients, and experimental models of podocyte and glomerular injury.

## 2. Methods

### 2.1. Study Approval and Ethics Statement

All research protocols and consent documents were approved by the Institutional Review Board of Nationwide Children’s Hospital as the coordinating center (approval numbers IRB07-00400, IRB12-00039 and IRB05-00544), as well as by each of the other participating centers of the PNRC (formerly the Midwest Pediatric Nephrology Consortium; MWPNC). Informed written consent (and assent, where appropriate) was obtained from the parents of all participants before samples were collected, in accordance with the Declaration of Helsinki.

### 2.2. Pediatric Nephrotic Syndrome Patients and Sample Collection

Pediatric NS patients aged between 18 months and 18 years were included in this study if they exhibited 3+ proteinuria and edema. Paired plasma samples were collected for each patient, with the first sample “Pre-treatment” at the time of disease presentation, and the second sample "Post-treatment" after ~7 weeks of GC therapy, when the clinical response of each patient to GC (i.e., SSNS or SRNS) was assessed and determined by the treating nephrologist. Table 1 describes the demographics of the patients used in this study. None of these patients were exposed to additional steroid sparing immunosuppressive therapies between the collection of our Pre- and Post-therapy samples, as is the standard of care for the treatment of childhood NS. Our primary selection criteria for RNA-seq sample selection were based on adequate high-quality sample availability. For assays performed on the larger cohorts, we used all the other available samples and segregated them into categories to distinguish those who were completely steroid-naïve vs. those exposed to steroids for varying numbers of days prior to the first sample collection. Please see Supplementary Material for details.

### 2.3. Sequencing, Analyses and Data Access

Total leukocyte RNA was extracted, RNA quality verified, and cDNA libraries were generated from 12 pediatric patients with either SSNS (*n* = 8) or SRNS (*n* = 4), according to the manufacturer’s instructions. These patient samples were selected based on availability of high-quality RNA for sequencing. RNAseq data from SSNS and SRNS patients (paired Pre and Post samples, from before and after GC treatment, respectively) were collected and the sequences were aligned to the International Human Genome Sequencing Consortium human reference sequence. We performed power analysis for RNA seq data using R package RNASeqPower and assumed a coefficient of variation of 0.4 for human samples [29]. With 8 samples in one group and 4 samples in the other, one can detect a 2.3-fold difference at 90% power in genes with coverage ≥100 reads. Mature mRNA reads derived from annotated sequences were included in this study, whereas all other expressed sequences were excluded. These read numbers of the samples collected before (Pre) and after (Post) GC treatment from 11 patients (one SSNS patient sample gave poor reads and was excluded from the study) were used to calculate gene expression ratios (GER). All these methods are detailed in the Supplementary Materials. The RNAseq data have been deposited in NCBI’s Gene Expression Omnibus [30] and are accessible through GEO Series accession number GSE145969 (https://www.ncbi.nlm.nih.gov/geo/query/acc.cgi?acc=GSE145969) as also mentioned in Data Sharing. The following secure token has been created to allow review of record GSE145969 while it remains in private status: mfexcaqunlmvlwn.

### 2.4. Selection of Candidate Genes Using Statistical Analyses

To identify a smaller set of genes that can discriminate between SSNS vs. SRNS patient groups, “volcano plotting” followed by hierarchical clustering method and scatter plotting were employed. These methods are detailed in the Supplementary Materials.

### 2.5. Selection of Candidate Genes Using the ICGA-ELM Algorithm

Following the statistical analyses, the Integer-Coded Genetic Algorithm (ICGA), based on the GA [18,31,32], and the neural network-based extreme learning machine (ELM) algorithm [18,33,34], jointly known as the ICGA-ELM algorithm, were used to obtain a small set of genes that reliably discriminated between the SSNS and SRNS patient groups as detailed in the Supplementary Materials.

### 2.6. Gene Expression Validation by qRT-PCR

qRT-PCR was performed for SULF1, SULF2, and RPL19 genes on the peripheral blood cDNA, which was prepared from patient whole-blood RNA as described in the supplementary materials.

### 2.7. Arylsulfatase Activity of SULF2 in the Plasma of Nephrotic Syndrome Patients

Arylsulfatase activity of SULF2 was measured in the NS patient plasma samples using a fluorescence assay, with slight modification of a previously described assay [35]. Please refer to the Supplementary Materials for details.

### 2.8. VEGF Binding Assay in the Plasma of Nephrotic Syndrome Patients

Endosulfatase activity of SULF2 in the Pre- and Post-treatment patient plasma was measured by indirect VEGF binding activity assay, as reported by Uchimura et al. [35], and described in detail in the Supplementary Materials.

### 2.9. VEGF Quantitation in the Plasma of Nephrotic Syndrome Patients

Total VEGF levels in the Pre- and Post-treatment patient plasma were measured by a commercial ELISA assay, according to manufacturer’s instructions, and the process is described in detail in the Supplementary Materials.

### 2.10. Podocyte Culture and Treatments, SULF2 mRNA Expression and VEGF Quantitation

Cultured human podocytes were differentiated, injured with PAN, and treated with dexamethasone. RNA was isolated to measure SULF2 expression and VEGF levels were quantitated in the culture media, as described in detail in the Supplementary Materials.

### 2.11. PAN-Induced Proteinuria Model and Glomerular SULF2 mRNA Expression

Male Wistar rats were injected with a single dose of PAN to induce nephropathy and proteinuria, as we have previously reported [36]. Glomeruli were isolated at Day 11 and RNA was isolated to measure the relative levels of SULF2 in control vs. nephrotic animals. See the Supplementary Materials for the detailed protocol.

### 2.12. Statistical Analyses

Statistical significance of the differences between SSNS vs. SRNS patient groups was set at *p* < 0.05. Ordinary one-way ANOVA was used for multiple comparisons among the SSNS Pre- vs. Post-treatment and the SRNS Pre- vs. Post-treatment groups. Unpaired Student’s *t*-tests were used for Post-treatment vs. Pre-treatment comparisons on the same patients. All the results are presented as mean values and SEM.

## 3. Results

### 3.1. Patient Demographics

Sixty-six pediatric patients were enrolled from 2008–2014, and 132 paired samples were collected as 1st and second samples, representing “Pre-treatment” and “Post-treatment” samples, as well as detailed clinical data (Table 1). Forty-six patients were clinically phenotyped as SSNS, since they achieved complete remission of proteinuria within an average of ~7 weeks of steroid therapy, while 20 patients did not achieve remission and were thus phenotyped as SRNS. Of the samples collected from the 66 patients, 45 (68%) of the first samples "Pre-treatment" were essentially steroid naïve. Since these were newly diagnosed cases of NS, none of these pediatric patients were exposed to additional steroid sparing immunosuppressive therapies between the collection of the Pre- and Post-therapy sample Twelve patients were subjected to transcriptomic analyses as a discovery cohort and up to a total of 66 patients were used as a validation cohort for subsequent analyses (Table 1). Cohort numbers available for the various studies of gene expression, protein activities, and plasma levels sometimes varied unavoidably due to constraints on patient samples and resources, and these are listed in the Supplementary Materials and in each Figure legend. Demographics comparisons between the SSNS and SRNS groups revealed that children with SSNS were different from those with SRNS in age, height, and weight. SRNS patients presented at a later mean age than those with SSNS (9.5 vs. 5.0 years; *p* = 0.0001), consistent with known mean ages of presentation for these different forms of NS. Although there were no gender differences, African Americans comprised a greater percentage of SRNS vs. SSNS patients (35% vs. 17%; *p =* 0.0009) in the total cohort of 66 patients.

### 3.2. Identification of Candidate Genes by RNASeq and Statistical Analysis

**Figure 1:**
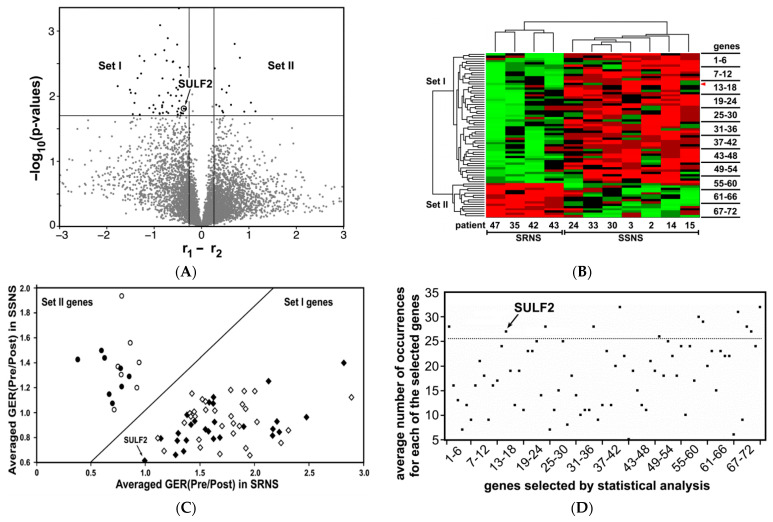
Identification of Candidate Genes by RNASeq, Statistical Analysis and ICGA-ELM Algorithm. The RNA-seq primary data set consisted of expression values for >20,000 genes in 11 out of 12 patients, which were reduced to 7334 genes by the removal of genes with little or no expression in one or several samples. These expression values were then used to calculate gene expression ratios (GER) before (Pre) and after (Post) treatment with glucocorticoids (GC) (GER(Pre/Post)). (**A**) “Volcano Plotting” of the gene expression ratios of 7734 genes following treatment with GC in nephrotic syndrome (NS) patients with steroid sensitive NS (SSNS) and steroid resistant NS (SRNS) identified a small set of putative marker genes. For each gene, the difference r_1_-r_2_ of the mean expression ratios GER(Pre/Post) of all samples of the SSNS patient group (r1) and of the SRNS patient group (r2) is plotted on the *X*-axis, and *t*-test criteria are plotted on the *Y*-axis. Given the selected thresholds (cut offs: *p*-value = 0.02 on the *Y*-axis; |r_1_-r_2_| = 0.263 on the *X*-axis), this plot identified 72 genes that were differentially expressed in the leukocytes of both groups of patients following treatment with GC (labeled as Set I and Set II in the upper left and right regions of the plot). The names of the 72 genes are listed in Table 2. SULF2 is among these 72 genes. (**B**) Clustering of the selected 72 genes according to their expression ratios results in an accurate grouping of the SRNS and SSNS patients. The 72 genes identified were hierarchically clustered, using their GER(Pre/Post). The cluster tree on the left defines Set I (genes 1–57) and Set II (genes 58–72) genes, whereas the cluster tree on the top separates the SRNS and SSNS patients. The GER(Pre/Post) of Set I and Set II genes perfectly partitioned the SRNS and SSNS patient groups. Up-regulated (green) and down-regulated (red) genes are marked, following treatment of the patients with GC. SULF2 is among the group of the Set I genes (red arrowhead at the top right of figure). The names of the 72 genes are listed in Table 2. (**C**) Linear separation of the expression ratios of the selected 72 genes in SRNS and SSNS patient groups suggested a high discriminatory potency. The GER(Pre/Post) for all patients were averaged for each of the selected 72 genes and plotted against each other with the ratios for the SSNS and SRNS patients shown on the *Y*-axis and *X*-axis, respectively. Set I genes (round symbols) and Set II genes (diamond symbols) formed separate clusters for both patient groups, which could be separated by a straight line (linear separation). SULF2 is labeled among the group of the Set I genes. The linear separation between these two gene groups suggested that their GER(Pre/Post) could be used to distinguish between SRNS and SSNS patients. The Integer-Coded Genetic Algorithm (ICGA) – Extreme Learning Machine (ELM) algorithm was then applied to identify smaller sets of genes which were able to distinguish between patient groups. Among them was a set of 34 genes which classified, with high accuracy, the SRNS and SSNS patient groups (closed symbols). The genes in this reduced gene set are also listed in Table 2. (**D**) The ICGA-ELM algorithm selected a small set of genes with high discriminatory potency. The GER(Pre/Post) of all 72 selected genes was analyzed by the ICGA-ELM algorithm. Hundreds of iterations of this algorithm produced several hundred sets of genes. Among these sets, a number of genes occurred with increased frequency. The twelve genes with the most frequent occurrences are found in the area above the dotted line and are listed in Table 3. SULF2 is among these top twelve genes.

To identify a small set of genes able to discriminate between patients with SSNS and SRNS, the gene expression ratios (GER) (Pre/Post) values obtained were initially analyzed by “volcano” plotting (see Methods). This representation filters out those genes with differential large-magnitude expression changes which are also statistically significant. Figure 1A graphically shows that 72 genes were differentially expressed above the selected thresholds. The genes are located in the upper left or upper right areas of the graph, indicating large absolute differences in the mean GERs and small *p* values, and suggesting that the distribution in both sets is different.

Treatment with GC resulted in both induced (Set I genes 1–57, as listed in Table 2) and reduced (Set II genes 58–72, as listed in Table 2) gene expression. These genes represent possible biomarkers to differentiate patients with SSNS from those with SRNS. Included among the Set I genes is SULF2, which has been reported to have a genetic association with NS, as well as to play a crucial role in the physiology of podocytes and in the maintenance of glomerular integrity [19,20,21].

To independently assess the discriminatory potential of these 72 genes, they were hierarchically grouped in a clustergram according to their GER(Pre/Post). The cluster tree on the left side of Figure 1B shows that the Set I genes form a cluster that is distinct from the cluster of the Set II genes. Clustering these genes clearly differentiated between children with SRNS and SSNS, as shown by the cluster tree at the top of the figure. Consistent with Figure 1A, Set I genes tended to have a higher GER(Pre/Post) in children with SRNS compared to those with SSNS, whereas Set II genes tended to exhibit the opposite response. The GER(Pre/Post) for SULF2 [GER(Pre/Post)_SULF2_] placed it in the group of Set I genes (red arrowhead). In addition, the two sets of genes (Set I and Set II) had excellent discriminatory power to differentiate SRNS from SSNS patients (Table 2).

The difference between the two patient groups is also evident by the linear separation of the averaged GER(Pre/Post) of the 72 genes (Figure 1A) when the values for SRNS and SSNS patients were plotted against each other (Figure 1C). This plot demonstrates a clear and linear separation of Set I (diamond symbols) and Set II (round symbols) genes, and thereby also of the SRNS and SSNS patients, suggesting a correlation between the two independent variables “GER(Pre/Post)” and “patient grouping”. Consistent with the results shown in Figure 1A,B, the averaged GER(Pre/Post)_SULF2_ falls into the group of Set I genes.

Taken together, the statistical methods applied identified 72 genes, including SULF2, that have the potential to discriminate between children with SSNS and SRNS, and thus may serve as candidate genes involved in GC resistance.

### 3.3. Identification of Candidate Genes by “Integer-Coded Genetic Algorithm” and “Extreme Learning Machine Algorithm”

To reduce the number of potentially discriminatory candidate genes, we then applied the Integer-Coded Genetic Algorithm (ICGA) in combination with the neural network-based Extreme Learning Machine (ELM) algorithm (i.e., the ICGA-ELM algorithm) to obtain the smallest set of twelve genes, which will give the maximum accuracy in distinguishing the two patient groups (SRNS vs. SSNS). Hundreds of iterations of ICGA-ELM runs identified several sets of genes which were able to distinguish the two patient groups with an overall accuracy of >95%, with all samples being correctly classified in all runs, except one SRNS sample that was incorrectly classified in a few runs.One of the sets of genes that classified both patient groups with high accuracy is indicated in Figure 1C (closed symbols) and is also listed in Table 2.

Among these smaller sets, a number of genes occurred more often than others. The average number of occurrences is shown in Figure 1D. The twelve genes with the most frequent occurrences, including SULF2, can be seen in the area above the dotted line, and are listed in Table 2 and their functions described further in Table 3.

### 3.4. SULF2 Gene Expression in SSNS vs. SRNS

**Figure 2:**
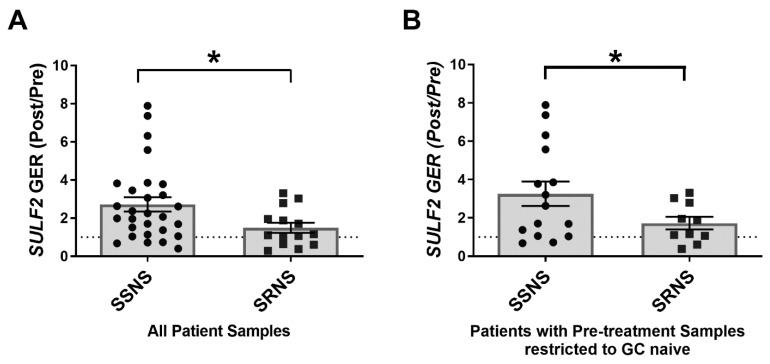
SULF2 Gene Expression in Children with steroid sensitive nephrotic syndrome (SSNS) vs. steroid resistant nephrotic syndrome (SRNS). cDNA synthesized from peripheral blood leukocyte RNA from SSNS and SRNS patients was used for determination of SULF2 expression. Gene expression ratios of SULF2 were determined by qRT-PCR in samples taken before and after an average of 7 weeks of glucocorticoid (GC) therapy (SULF2 GER(Post/Pre)). (**A**) The SULF2 GER(Post/Pre) was significantly greater in samples from SSNS (*n* = 28) vs. SRNS (*n* = 14) patients (2.72 ± 0.37 SEM vs. 1.49 ± 0.26 SEM; *p* = 0.0358 by unpaired two-tailed *t*-test, * denotes significance). The dotted line marks a SULF2 GER(Post/Pre) = 1, which separates induced SULF2 expression (above the line) from reduced (below the line) SULF2 expression following GC treatment. Since some of these patients received one or more GC doses prior to the pre-treatment sample collection, the data were further segregated based on GC exposure. (**B**) Patients were included only if they were completely GC naïve with confirmation of no GC exposure prior to collection of the pre-treatment sample. In this near-ideal clinical research setting the SULF2 GER(Post/Pre) was still greater in samples from SSNS (*n* = 15) vs. SRNS (*n* = 10) patients (3.26 ± 0.64 SEM vs. 1.72 ± 0.33 SEM; *p* = 0.0449 by unpaired two-tailed *t*-test with Welch’s correction, * denotes significance).

To evaluate and validate whether SULF2 expression is discriminatory between patients with SSNS and SRNS, we measured its expression before (Pre) and after (Post) an average of ~7 weeks of GC therapy by qRT-PCR in leukocytes from a larger validation cohort of children with SSNS and SRNS (Figure 2). The GER(Post/Pre)_SULF2_ was found to be significantly greater in samples from SSNS vs. SRNS patients (2.72 vs. 1.49, *p* = 0.0358) (Figure 2A). When only steroid naïve patients were analyzed, the GER(Post/Pre)_SULF2_ was still greater in SSNS vs. SRNS patients (3.26 vs. 1.72, *p* = 0.0449) (Figure 2B).

### 3.5. Plasma Arylsulfatase Activity in SSNS vs. SRNS

**Figure 3:**
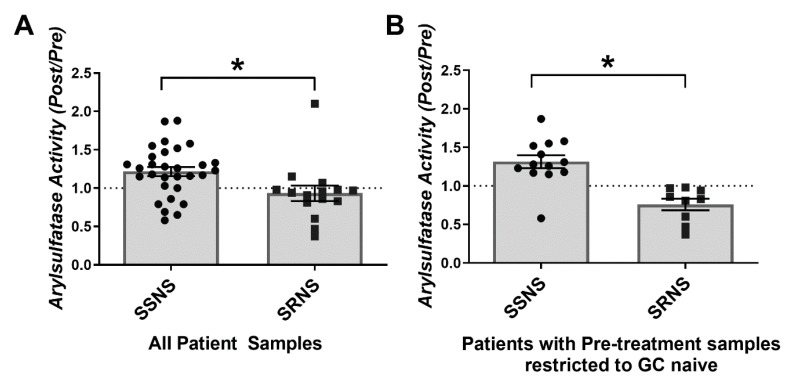
Plasma arylsulfatase activity in children with steroid sensitive nephrotic syndrome (SSNS) vs. steroid resistant nephrotic syndrome (SRNS). Clarified Pre-treatment and Post-treatment plasma samples from SSNS and SRNS patients were used for determinations of arylsulfatase activity. The results are plotted as ratios of the activities in the two samples collected from each patient after glucocorticoid (GC) treatment vs. before GC treatment (Post/Pre). (**A**) The arylsulfatase activity (Post/Pre) was significantly greater in samples from SSNS (*n* = 30) vs. SRNS (*n* = 15) patients (1.22 ± 0.06 SEM vs. 0.93 ± 0.10 SEM; *p* = 0.0135 by unpaired two-tailed *t*-test, * denotes significance). The dotted line marks where the arylsulfatase activity ratio = 1, which separates increased (above) from reduced (below) enzyme activity after GC therapy. Since some of these patients received one or more GC doses prior to the pre-treatment sample collection, the data were further segregated based on GC exposure. (**B**) Patients were included only if they were completely GC naïve with confirmation of no GC exposure prior to collection of the pre-treatment sample. In this near-ideal clinical research setting the arylsulfatase activity (Post/Pre) still remained significantly greater in samples from SSNS (*n* = 13) vs. SRNS (*n* = 9) patients (1.31 ± 0.08 SEM vs. 0.76 ± 0.07 SEM; *p* = 0.0001 by unpaired two-tailed *t*-test, * denotes significance).

The *SULF2* protein has arylsulfatase activity at pH 8.0 in addition to endosulfatase activity, which can be used for enzymatic measurements. This arylsulfatase activity was measured in clarified Pre- and Post-GC treatment plasma samples from SSNS and SRNS patients and the results are presented as activity ratios (Post/Pre) (Figure 3). The plasma arylsulfatase activity (Post/Pre) was significantly greater in samples from SSNS vs. SRNS patients (1.22 vs. 0.93, *p* = 0.0135) (Figure 3A). When these data were analyzed in the steroid-naïve cohort only (Figure 3B), the plasma arylsulfatase activity (Post/Pre) remained significantly greater in samples from SSNS vs. SRNS patients (1.31 vs. 0.76, *p* = 0.0001). Thus, consistent with the SULF2 gene expression data shown in Figure 2, plasma arylsulfatase activity ratios were significantly greater in plasma samples from children with SSNS vs. SRNS. Of note, while plasma arylsulfatase activity decreased in a majority of SRNS patients after GC therapy, it increased in a majority of SSNS patients. More importantly, the arylsulfatase activity ratio was significantly different in SSNS vs. SRNS patients regardless of initial exposure to steroids in some of these NS patients, which is important as the vast majority of patients present in the nephrology clinic having received at least one prior dose of steroids.

Since the biochemical assay for arylsulfatase activity cannot differentiate between SULF2 and SULF1, we also measured the *SULF1* mRNA expression in the patients’ peripheral leukocytes and compared it with the expression of *SULF2*, using semi-quantitative RT-PCR (Figure S1**)**. While robust signals for the expression of both SULF2 and the control gene RPL19 were observed, we were unable to detect SULF1 expression either before or after GC therapy. Thus, leukocyte SULF1 is unlikely to contribute to the plasma arylsulfatase activity shown in Figure 3.

### 3.6. Plasma VEGF Binding Activity in SSNS vs. SRNS

**Figure 4:**
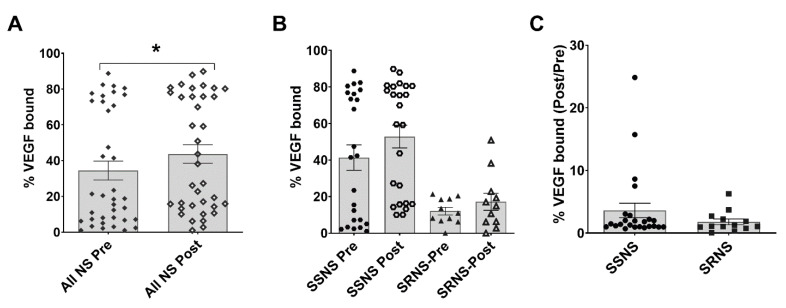
Plasma Endosulfatase Activity (Measured by Vascular Endothelial Growth Factor [VEGF] Binding to Heparin) in Children with steroid sensitive nephrotic syndrome (SSNS) vs. steroid resistant nephrotic syndrome (SRNS). Glucocorticoid (GC) naïve pre-treatment and post-treatment plasma samples from SSNS (*n* = 25) and SRNS (*n* = 13) patients were used to determine the effects on binding of VEGF to immobilized heparin as an indirect measure of plasma endosulfatase activity. (**A**) The mean % VEGF bound was significantly increased after GC treatment in all patients combined (*n* = 24 SSNS pts + *n* = 13 SRNS pts) (Pre: 34.44 ± 5.28 SEM vs. Post: 43.68 ± 5.20 SEM; *p* = 0.0005 by paired two-tailed *t*-test, * denotes significance). (**B**) The mean % VEGF bound tended to be higher in SSNS patients (Pre: 41.36 ± 6.97 SEM, Post: 52.83 ± 6.22 SEM) compared to SRNS patients (Pre: 21.66 ± 6.97 SEM, Post: 26.78 ± 7.55 SEM), but did not differ significantly amongst the four cohorts, as determined by ordinary one-way ANOVA analysis. (**C**) Similarly, the mean Post/Pre % VEGF bound ratios were not significantly different between SSNS vs. SRNS patients, as determined by unpaired t tests (3.61 ± 1.15 SEM vs. 1.79 ± 0.46 SEM; *p* = 0.2695).

SULF2 also has endosulfatase activity, which can be measured by the modulation of VEGF binding to immobilized heparin in an ELISA assay, as described earlier [35]. This endosulfatase activity was measured in steroid naïve Pre- and Post-GC treatment plasma samples from SSNS and SRNS patients and the results are presented as “% VEGF bound” values plotted against all Pre- vs. Post-treatment samples, different cohorts, and as ratios in the two samples collected from each SSNS or SRNS patient (Post/Pre) (Figure 4). The % VEGF bound was significantly increased in all plasma samples after GC treatment from the SSNS (*n* = 24 pts) and SRNS (*n* = 13 pts) patients combined (Pre: 34.44 vs. Post: 43.68, *p* = 0.0005) (Figure 4A). While the % VEGF bound tended to be higher in SSNS (Pre: 41.36, Post: 52.83) vs. SRNS samples (Pre: 21.66, Post: 26.78), it did not differ significantly amongst the four cohorts (Figure 4B). Similarly, the Post/Pre % VEGF bound ratio comparisons did not show a significant difference between SSNS vs. SRNS samples (3.61 vs. 1.79, *p* = 0.2695) (Figure 4C). Of note, all the patient pre-treatment samples used in this VEGF binding analysis were steroid-naïve.

### 3.7. Plasma Quantitative VEGF Levels in SSNS vs. SRNS

**Figure 5:**
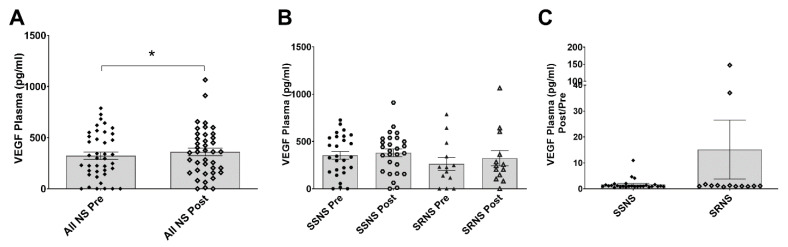
Plasma quantitative vascular endothelial growth factor (VEGF) levels in children with steroid sensitive nephrotic syndrome (SSNS) vs. steroid resistant nephrotic syndrome (SRNS). Glucocorticoid (GC) naïve pre-treatment and post-treatment plasma samples from SSNS (*n* = 27) and SRNS (*n* = 13) patients were used to measure plasma VEGF levels. (**A**) The VEGF levels were significantly increased in all samples after GC treatment in all patients combined (*n* = 27 SSNS pts + *n* = 13 SRNS pts) (Pre: 324.10 ± 36.06 SEM vs. Post: 361.70 ± 37.63 SEM, *p* = 0.0340 by paired two-tailed *t*-test, * denotes significance). (**B**) The VEGF levels did not differ significantly amongst the four cohorts as determined by ordinary one-way ANOVA analysis (SSNS Pre: 345.00 ± 41.79 SEM, SSNS Post: 380.30 ± 40.71 SEM, SRNS Pre: 262.00 ± 68.34 SEM, SRNS Post: 323.30 ± 80.74 SEM; *p* = 0.4957 summarized ANOVA). (**C**) Similarly, the Post/Pre VEGF level ratios were not significantly different between SSNS vs. SRNS patients, as determined by unpaired *t* test (1.72 ± 0.40 SEM vs. 15.14 ± 11.34 SEM; *p* = 0.0927).

Total VEGF levels in the plasma of Pre- and Post-GC treatment samples of 27 SSNS and 13 SRNS patients were measured, analyzed and the results are presented as VEGF levels (pg/mL) plotted against all Pre- vs. Post-GC treatment samples, different cohorts, and as ratios in the two samples collected from each SSNS or SRNS patient (Post/Pre) (Figure 5). The overall plasma VEGF levels were significantly increased in all samples after GC treatment from the SSNS (*n* = 27 pts) and SRNS (*n*= 13 pts) patients combined (Pre: 324.10 vs. Post: 361.70, *p* = 0.0340) (Figure 5A). The plasma VEGF levels did not differ significantly amongst the four cohorts (SSNS Pre: 345.00, SSNS Post: 380.30, SRNS Pre: 262.00, SRNS Post: 323.30) (Figure 5B). Similarly, the Post/Pre VEGF level ratio comparisons did not show a significant difference between SSNS vs. SRNS (1.72 vs. 15.14, *p* = 0.0927) (Figure 5C). Of note, all the patient pre-treatment samples used in this analysis were steroid-naïve.

### 3.8. SULF2 Gene Expression in Nephrotic Glomeruli and Injured Podocytes

**Figure 6:**
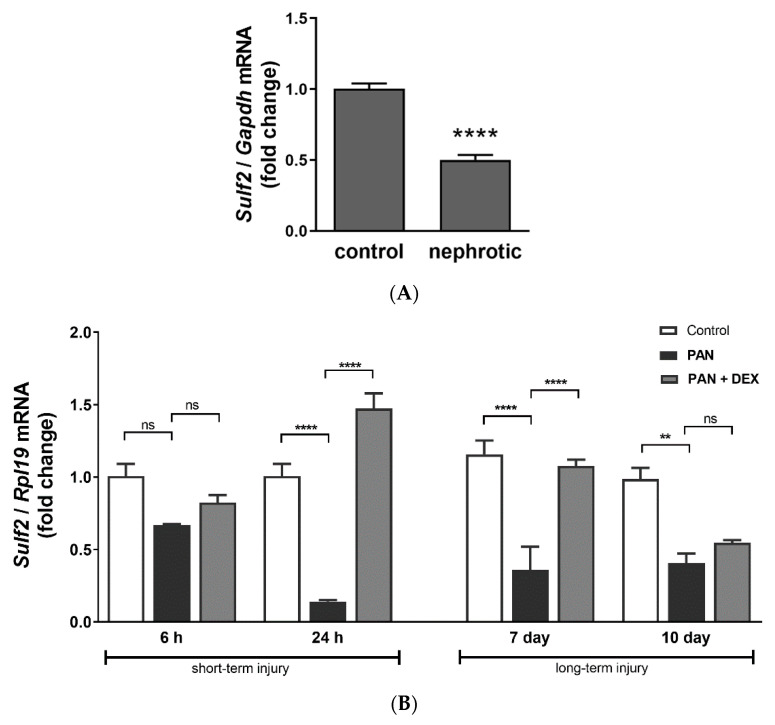
SULF2 Gene Expression in Nephrotic Glomeruli and Injured Podocytes. (**A**) Glomeruli were isolated from puromycin aminonucleoside (PAN)-induced nephrotic rats on Day 11 after PAN injection, when they showed significant proteinuria, and RNA processed for the determination of SULF2 gene expression. Glomerular SULF2 gene expression was significantly decreased (50%) in nephrotic rats vs. Controls (*n* = 3 rats/group) as determined by unpaired *t* test (Control: 1.00 ± 0.04 SEM vs. Nephrotic: 0.50 ± 0.03 SEM; *p* < 0.0001). (**B**) Cultured podocytes were exposed to PAN for 6 h, 24 h, 7d and 10d to determine the time course of podocyte SULF2 gene expression after PAN-induced injury. Podocyte SULF2 gene expression was significantly reduced in a short-term injury model with high PAN concentration after 24 h (1.01 ± 0.08 SEM vs. 0.14 ± 0.01 SEM; *p* < 0.0001), but not after 6 h (1.01 ± 0.08 SEM vs. 0.67 ± 0.00 SEM, *p* = 0.3254, ns). Podocyte SULF2 gene expression was also significantly reduced in a longer-term injury model with low PAN concentration after both 7 d (1.16 ± 0.09 SEM vs. 0.36 ± 0.16 SEM; *p* < 0.0001) and 10 d (0.99 ± 0.08 SEM vs. 0.41 ± 0.06 SEM; *p* = 0.0017), as determined by one-way ANOVA analysis of multiple comparisons. Notably, treatment of PAN-injured podocytes with GC (i.e., dexamethasone (DEX)) partially reversed the reductions in SULF2 gene expression after both 24 h and 7d injury with PAN (24 h: 0.14 ± 0.01 SEM vs. 1.47 ± 0.10 SEM; *p* < 0.0001; 7 d: 0.36 ± 0.16 SEM vs. 1.08 ± 0.04 SEM; *p* < 0.0001). (**** *p* < 0.0001; ** *p* < 0.005; ns, *p* > 0.05.)

Due to the recognized protective role of sulfatase in glomeruli and podocytes [19,20,21,47], we studied its glomerular expression in an animal model of NS as well as in cultured podocytes. While we recognize that the plasma and glomerular SULF2 expression and downstream activities are unlikely to be directly linked, given its molecular activity, we remained very interested in SULF2’s overall effects and regulation in NS. Puromycin aminonucleoside (PAN)-induced nephropathy resulted in significant proteinuria in male Wistar rats on Day 11, as we have previously reported [36]. In glomeruli isolated from these rats, we found that SULF2 gene expression was significantly reduced (50%) in nephrotic rats vs. Controls (*n* = 3 rats/group) (Control: 1.00 vs. Nephrotic: 0.50, *p* < 0.0001) (Figure 6A). Cultured differentiated podocytes also revealed a similar pattern of reduced SULF2 gene expression upon injury with PAN. SULF2 expression was reduced in a short-term podocyte injury model with high PAN concentration after 24 h (1.01 vs. 0.14, *p* < 0.0001), but not after 6 h (1.01 vs. 0.67, *p* = 0.3254, ns) as well as in a longer-term injury model with low PAN concentration after 7 d (1.16 vs. 0.36, p ≤ 0.0001) and 10 d (0.99 vs. 0.41, *p* = 0.0017) (Figure 6B). Interestingly, the treatment of PAN-injured podocytes with GC (i.e., dexamethasone; DEX) reversed the reduction in SULF2 gene expression, after both the 24 h and 7 d PAN-induced injury models (24 h: 0.14 vs. 1.47, *p* < 0.0001; 7 d: 0.36 vs. 1.08, *p* < 0.0001) (Figure 6B). We also measured the release of VEGF-A into the culture media from cultured podocytes injured with PAN and treated with dexamethasone using an ELISA assay, to assess any association with SULF2 expression (see Supplementary Materials). While we observed a decrease in VEGF in the media after podocyte injury, this decrease seemed to be mostly attributed to cell death, as normalization of these VEGF levels to the total cellular protein content revealed no significant differences among the groups (Figure S2).

## 4. Discussion

Nephrotic syndrome is among the most common kidney diseases seen in both children and adults [1]. The primary therapy for NS, oral GCs, induces remission in most patients, although ~10–20% of children and ~50% of adults with NS either present with or develop GC resistance later in their course [2,3]. Moreover, the molecular basis for clinical resistance to GC therapy remains largely unknown [1,2,7]. In the current study, we performed transcriptome-wide gene sequencing and analysis of circulating leukocytes from patients with SSNS and SRNS to better understand the molecular basis of steroid sensitivity vs. resistance in NS. The peripheral blood leukocytes were collected both at clinical presentation with NS and after ~7 weeks of GC therapy from children with either SSNS or SRNS recruited through the PNRC. We identified a gene panel able to differentiate SRNS from SSNS using in silico algorithm-based approaches of the RNA seq data and further studied one of the relevant genes identified in the 12-gene panel, SULF2, which encodes an extracellular endoglucosamine-6-sulfatase that has been previously reported to play crucial roles in podocyte physiology and the maintenance of glomerular integrity, both of which are critically affected in NS [19,20,21]. Subsequent analyses of SULF2 levels and activity in SRNS and SSNS patient plasma, as well as in experimental models of NS and podocyte injury, revealed that clinical steroid resistance in childhood NS was associated with lack of increases in both SULF2 leukocyte expression and plasma activity after GC therapy, and that SULF2 expression was downregulated in experimental models of both podocyte and glomerular injury. These findings together suggest that SULF2 may play a protective role in NS, independent of plasma VEGF regulation, and thus that SULF2 manipulation could potentially be exploited for the development of novel future treatments for NS.

NS is recognized as a systemic disease but is now also known to be characterized by direct podocyte injury [1,4,5,6]. RNAseq from blood cells can provide valuable information about this disease as it captures the differential expression of genes from circulating cells and is far less invasive than a renal biopsy. This study was not designed to identify biomarkers predictive of steroid resistance, but rather a mechanistic understanding of molecular pathways with potential roles in regulating steroid resistance, as patient samples were assessed both before and after steroid therapy. Due to biological variability, a larger patient cohort would be necessary to identify and validate predictive or associative biomarkers of steroid resistance using a discovery approach. Our study identified twelve candidate genes that, when evaluated together, have high discriminatory accuracy to distinguish patients with SSNS from those with SRNS (Table 3). While the present study focuses specifically on the identification and important role of SULF2 in NS, the molecular basis for the discriminatory potency of the other 11 identified genes in clinical steroid resistance in NS remains to be elucidated in future studies.

The human genome encodes 17 members of the sulfatase superfamily [24]. Among them, SULF2 and its paralog, SULF1, encode proteins that are distinguished by both their extracellular location and their endo-sulfatase activity that strictly requires a neutral pH, as is found in the extracellular space [22,24]. Human gene expression data demonstrate SULF2 expression in many adult tissues (http://biogps.org/#goto=genereport&id=55959). SULF2 is expressed in lymphocytes, monocytes and neutrophils, as well as other tissues in the body, such as endometrium, breast epithelial cells, adipose tissue and kidney cortex. The Immunological Genome Project database also reports that monocytes are the main category of immunological cells expressing SULF2 in blood (http://rstats.immgen.org/Skyline/skyline.html). SULF2 can be released from cells and its expression has been detected in various normal tissues as well as in healthy blood. The expression of SULF2 has also been reported in the plasma and serum of healthy individuals from peripheral blood mononuclear cells, and the overall plasma levels have been shown to be increased in cirrhosis [48]. SULF2 is synthesized as a preproprotein (870 aa) with a cleavable signal peptide, an amino-terminal region containing the catalytic site, a hydrophilic domain (HD), and a C-terminal region. After the removal of the signal peptide, the proprotein (125 kDa) is cleaved within the HD domain by furin-related proteinases into a 75 kDa amino-terminal fragment, and a 50 kDa C-terminal fragment [49]. The 75 kDa and 50 kDa subunits are then joined by disulfide bonds to form a heterodimer. The heterodimer, as well as the 125 kDa proprotein both exhibit endosulfatase activity against heparan sulfate [49]. In SULF2 transfected cells, fully processed and enzymatically active forms can be found in the conditioned medium, as well as on the cell surface [22,49].

Both SULF2 and SULF1 exhibit the same substrate specificity, and thus cannot be distinguished biochemically [50]. The other sulfatases are located in lysosomes and have exo-sulfatase activities on proteoglycan chains [51], or are located in the endoplasmic reticulum and the Golgi apparatus [52]. Thus, enzymatic assays at a neutral or slightly alkaline pH that use extracellular liquids (e.g., plasma) capture only SULF2 and SULF1 activity and exclude the other sulfatases. Both SULF2 and SULF1 seem to play a crucial role in kidney development and function. In mice, SULF2 expression was found in the nephron progenitor cells and tubules, and SULF1/SULF2 double knock-out mice suffered from kidney hypoplasia [19]. SULF2 but not SULF1 knock-out mice exhibited an increase in UA2S-GlcNS6S in kidneys, supporting a function in these organs [23]. Importantly, negatively charged heparan sulfate proteoglycans, and in particular their degree of sulfatation in the glomerular basement membrane, play a major role in glomerular filtration [53,54]. Consistent with this, mice with deleted SULF1 and SULF2 genes developed proteinuria resulting from injury of both glomerular endothelial cells and podocytes [19]. Further support for the connection between SULF2/SULF1 and proteinuria was obtained from children with Wilms’ tumor. The largely podocyte-specific transcription factor Wilms’ tumor gene 1 (WT1) regulates the expression of SULF2 and SULF1, and children with this tumor exhibited a kidney phenotype similar to that in SULF2 and SULF1 knock-out mice [19]. Moreover, another study has identified a SNP association (rs11086243; upstream of SULF2) with NS [47]. In addition, a recent study reported critical roles for SULF1 and SULF2 in the maintenance of glomerular integrity and their protective roles in diabetic nephropathy [21]. Furthermore, a very recent study also identified critical roles for sulfatases in the maintenance of glomerular integrity using Zebrafish as a model [20], while a parallel study negated the role of heparan sulfate [55].

The protective roles of sulfatase in glomeruli and podocytes have been reported in a few studies [19,20,21,47]. Our animal and cell culture studies further support these prior studies associating markers of glomerular and podocyte injury, such as reduced synpatopodin and nephrin with the decreased expression of SULF2 [36]. Additionally, sulfatase activity has been reported to affect the binding and availability of growth factors, such as VEGF in glomeruli (podocytes and endothelial cells) [28,56,57]. While the current studies do not directly link glomerular SULF2 activity to plasma SULF2 activity, our findings do confirm an association between both plasma SULF2 activity and levels in clinical steroid resistance in childhood NS. These studies also suggest an important potential mechanistic role of sulfatase in the plasma that appears to be distinct from or in addition to the expected downstream VEGF regulation. Additional support for this possibility includes an ambiguous association between plasma VEGF levels and NS, resulting from conflicting results in prior studies [58], as well as that therapeutic inhibition of VEGF signaling has been associated with nephrotoxicity [59]. Notably, while we were not able to demonstrate differences in VEGF binding and levels between SSNS vs. SRNS, we observed a significant increase in both VEGF binding, as well as levels in combined NS group (Figure 4A and Figure 5A). Although not significant, we also observed a trend towards increased VEGF binding (Figure 4B) and levels (Figure 5B) in SSNS patients (Pre and Post) and it is possible that the measurements of these parameters would lead to significant differences in study with larger patient cohort.

**Figure 7:**
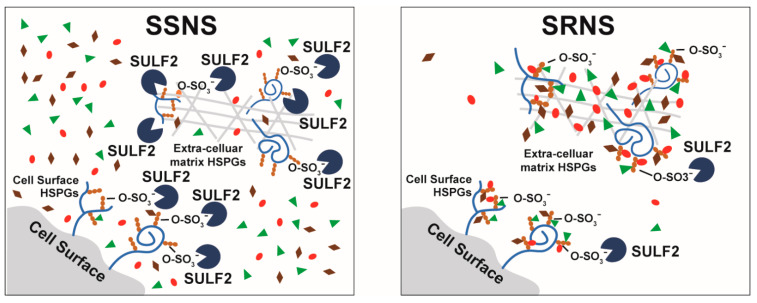
Schematic for GC-Induced Changes in SULF2 Expression and Activity in SSNS vs. SRNS. SULF2 modulates the sulfatation pattern on heparan sulfate proteoglycans (HSPGs) by removing 6-O-sulfates. HSPGs are found on the cell surfaces and in extracellular matrix where they interact with multiple ligands such as growth factors (VEGF, PDGF and FGF), chemokines and morphogens. By regulating the sulfatation pattern on HSPGs, SULF2 modulates the availability and activity of these ligands. Our data suggest that SSNS have higher SULF2 leukocyte expression as well as activity in the plasma of patients after GC therapy compared to SRNS. This would potentially result in increased cleavage of sulfate groups from HSPGs in SSNS and release of growth factors, chemokines and morphogens. However, the SULF2 endosulfatase activity as measured by VEGF-heparin binding as well as total VEGF plasma levels were not discriminatory in SSNS vs. SRNS. Our findings together suggest that SULF2 may play a protective role in NS, independent of plasma VEGF regulation, and thus SULF2 manipulation could potentially be exploited for the development of novel treatments for NS. 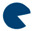, SULF2, Ligands (growth factors, 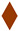; chemokines, 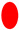; 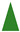 morphogens).

Heparan sulfate proteoglycans (HSPGs) are complex unbranched carbohydrate chains that conjugate with proteins on the cell surface or in the extra cellular matrix. HSPGs are highly sulfated and act as co-receptors for various molecules such as growth-factors, chemokines and morphogens. SULF2 remodels the 6-O-sulfatation of the cell’s surface or extra-cellular matrix HSPGs, thus regulating the activity and availability of the growth factors, chemokines and morphogens in their environment, has consequences for the modulation of transmembrane signaling processes (Figure 7). Upon desulfatation, extracellular ligands can no longer bind to the cell membrane or matrix [23]. Examples of affected signaling pathways, in addition to the interaction of VEGF-160, include the modulation of FGF-1 binding with heparin or heparan sulfate, and the promotion of Wnt/β-catenin signaling [45]. These other signaling pathways in the plasma, known to be affected by SULF2, could also potentially have mechanistic associations with steroid resistance in NS, although this is only speculative at this point. While we did not observe differences in VEGF binding or plasma levels between children with SSNS vs. SRNS, it is entirely possible that other ligands could potentially be affected by such differences in SULF2, and affect various other physiological processes, such as proliferation, apoptosis, differentiation, angiogenesis, immune invasion, matrix remodeling, cell adhesion, immune cell infiltration, etc. [60]. Indeed, important roles for HSPGs and their modifying enzymes such as SULF2 are increasingly being recognized in various pathophysiological process, and have already been identified as potential therapeutic targets in diseases such as cancer [60].

This study had several limitations and strengths. We are aware that some of the children with SRNS may have had an underlying genetic cause of disease. However, since these samples were collected over the previous decade, there were no IRB or financial provisions at that time to screen the patients for monogenic causes of disease. Despite this limitation, this study identifies a potentially important mechanistic role for sulfatase 2 in steroid resistance in NS, regardless of any possible underlying genetic mutations. In addition, our small discovery cohort for the identification of transcriptomic predictive biomarkers of SRNS was not ideal for predictive biomarker analysis, as we unfortunately were limited in our ability to utilize a transcriptomics approach for a larger cohort due to both resources and sample availability and quality (plasma vs. good quality RNA). We have, however, attempted to identify predictive biomarkers for SRNS in childhood NS in our recently published manuscripts using proteomics, metabolomics and cytokine profiling approaches on plasma from a much larger cohort of patient samples [61,62,63]. Nevertheless, our discovery cohort in the present study clearly identified a gene panel able to distinguish between patients with SSNS and SRNS (Pre and Post therapy) which enabled us to perform more definitive SULF2 expression and function assays in a larger cohort of patients, as well as assays in animal and cultured podocyte models of NS-related injury. Furthermore, while we observed age, weight and race differences between the SSNS and SRNS groups, there are no available data suggesting differences in SULF2 expression could be due to differences in age or race. Lastly, our studies cannot rule out the possibility that the identified relationship between sulfatase 2 and SRNS is associative rather than causal, because of the study design.

A major strength of this study was the evaluation of paired samples from each patient presenting with NS, both prior to initial steroid treatment and following an average of ~7 weeks of daily oral steroids when they had been declared to have either SSNS or SRNS by their treating pediatric nephrologist. This approach enabled us to analyze the differences between two clinically distinct cohorts of children with NS, both before and following their initial course of GC treatment. Additionally, SULF2 expression and activity was also measured and found to be distinct between SSNS vs. SRNS.

The present study is in line with current efforts to develop a comprehensive and integrated systems biology approach to diagnostic, prognostic, and therapeutic decisions related to NS, and to identify functional subgroups through the integration of clinical, histological, genetic, and molecular profiles. We have utilized RNA-seq in conjunction with additional innovative statistical methods and identified SULF2 as a primary gene of interest. An analysis of SULF2 gene expression and measures of plasma activity further confirmed a lack of increase in SULF2 leukocyte expression and plasma activity in patients with SRNS. Furthermore, SULF2 gene expression was also reduced in both rat glomeruli and cultured podocytes following NS-related injury.

In conclusion, the current studies revealed that clinical steroid resistance in childhood NS is associated with lack of increases in both SULF2 leukocyte expression and plasma activity after GC therapy, without apparent alterations in downstream VEGF plasma levels. Additionally, SULF2 expression was downregulated in experimental models of both podocyte and glomerular injury. These findings together suggest that SULF2 may play a protective role in NS, independent of plasma VEGF regulation, and thus that SULF2 manipulation could potentially be exploited for the development of novel future treatments for NS.

## 5. Translational Statement

The molecular basis for steroid resistant NS (SRNS) remains unclear. This study identified SULF2 among a panel of genes that distinguished SRNS from steroid-sensitive NS. SRNS correlated with lack of increases in SULF2 leukocyte expression and plasma activity after treatment, and SULF2 was downregulated in both NS models and podocyte injury. Moreover, despite VEGF’s role in mediating glomerular SULF2, no such correlation was evident in NS plasma. Thus, SULF2 may play a protective role in NS via the modulation of mediators distinct from VEGF, suggesting that SULF2 manipulation could potentially be exploited for the development of novel treatments for NS.

## Figures and Tables

**Table 1:** Patient demographics for children with steroid resistant nephrotic syndrome (SRNS) and steroid-sensitive nephrotic syndrome (SSNS).

	Discovery				Validation			
	Total	SSNS	SRNS	*p* Value	Total	SSNS	SRNS	*p* Value
*N*	12	8	4		66	46	20	
Weeks between Pre and Post GC treatment	5.9 ± 0.4, *n* = 12	6.0 ± 0.5, *n* = 8	5.6 ± 0.7, *n* = 4	ns	7.1 ± 0.4, *n* = 66	7.1 ± 0.4, *n* = 46	7.3 ± 0.6, *n* = 20	ns
Steroid-naïve at sample collection	10 (83%)	6 (75%)	4 (100%)		45 (68%)	30 (65%)	15 (75%)	
Steroid treatment 1 day prior to sample collection	2 (17%)	2 (25%)	_		8 (12%)	5 (11%)	3 (15%)	
Steroid treatment 1–3 days prior to sample collection	_	_	_		8 (12%)	7 (15%)	1 (5%)	
**Age**	6.5 ± 1.2, *n* = 12	4.0 ± 0.7, *n* = 8	11.5 ± 1.3, *n* = 4	0.0002 *	6.4 ± 0.5, *n* = 64	5.0 ± 0.5, *n* = 44	9.5 ± 0.8, *n* = 20	0.0001 *
Not Reported	_	_	_		2	2	_	
**Sex**								
Male	5 (42%)	4 (50%)	1 (25%)	0.0001 ^‡^	29 (45%)	21 (48%)	8 (40%)	ns
Female	7 (58%)	4 (50%)	3 (75%)		35 (55%)	23 (52%)	12 (60%)	
Not Reported	_	_	_		2	2	_	
**Height**	122.3 ± 7.9, *n* = 12	105.5 ± 4.4, *n* = 8	155.8 ± 5.5, *n* = 4	0.0001 *	119.9 ± 3.3, *n* = 63	110.9 ± 3.3, *n* = 44	140.7 ± 5.5, *n* = 19	0.0001 *
Not Reported	_	_	_		3	2	1	
**Weight**	38.8 ± 8.7, *n* = 12	20.6 ± 1.3, *n* = 12	75.2 ± 12.9, *n* = 4	0.0001 *	32.9 ± 2.8, *n* = 64	24.6 ± 2.0, *n* = 44	51.1 ± 6.2, *n* = 20	0.0001 *
Not Reported	_	_	_		2	2	_	
**Race**		SSNS	SRNS ^†^			SSNS	SRNS ^†^	
White	6 (50%)	5 (63%)	1 (25%)		29 (46%)	20 (43%)	9 (45%)	
Asian	2 (17%)	2 (25%)	0 (0%)		8 (12%)	8 (17%)	0 (0%)	
African American	3 (25%)	0 (0%)	3 (75%)	0.0001 ^†^	15 (23%)	8 (17%)	7 (35%)	0.0009 ^†^
Biracial	0 (0%)	0 (0%)	0 (0%)		3 (5%)	2 (4%)	1 (5%)	
Native American	0 (0%)	0 (0%)	0 (0%)		2 (3%)	1 (2%)	1 (5%)	
Not Reported	1 (8%)	1 (12%)	0 (0%)		9 (14%)	7 (15%)	2 (10%)	

* Unpaired *t* test, two tailed; **^†^** Chi-square test; ^‡^ Binomial test.

**Table 2:** Candidate genes identified by various bioinformatics approaches.

(a)									
#	Gene	Gene Set	Selected by ICGA-ELM	Selected by ICGA-ELM	#	Gene	Gene Set	Selected by BICGA-ELM	Selected by ICGA-ELM
		(Figure 1A–C)	(Figure 1C)	(Figure 1D)			(Figure 1A–C)	(Figure 1C)	(Figure 1D)
1	*RFFL*	I	X	X	37	*ZC3H12D*	I	X	
2	*INPP1*	I			38	*PRR5L*	I		
3	*MCM2*	I			39	*UBE2J2*	I		
4	*CSNK1G1*	I			40	*TOPBP1*	I	X	X
5	*SIGLEC10*	I			41	*GALNT2*	I	X	
6	*AGPAT6*	I	X		42	*BTN2A2*	I		
7	*DCAF8*	I			43	*GPR108*	I		
8	*FAM193A*	I			44	*CHMP4B*	I	X	
9	*RSPRY1*	I			45	*ALDOA*	I		
10	*KCTD20*	I			46	*TRAPPC1*	I	X	
11	*BLOC1S3*	I			47	*SPRYD3*	I	X	
12	*TOE1*	I			48	*MTMR14*	I	X	
13	*ACAD10*	I			49	*S1PR5*	I	X	X
14	***SULF2***	I	X	X	50	*FAM134C*	I		
15	*RAB8A*	I			51	*ALDOC*	I		
16	*NCRNA00294*	I			52	*TCP1*	I		
17	*ZNF318*	I			53	*CLCN3*	I		
18	*CDK4*	I			54	*NDUFS1*	I	X	
19	*TMEM219*	I	X		55	*LGALS9*	I	X	
20	*PRKCSH*	I	X		56	*SUPT5H*	I	X	
21	*SLC9A7*	I			57	*JARID2*	I	X	
22	*LEO1*	I			58	*FAM40A*	II		X
23	*STX4*	I	X	X	59	*KLF2*	II	X	X
24	*PTPRK*	I			60	*AKIRIN1*	II	X	
25	*GANAB*	I	X		61	*ZNF782*	II		
26	*C17orf63*	I			62	*OCIAD1*	II	X	
27	*ZC3H18*	I	X		63	*KPNA1*	II		
28	*MED14*	I			64	*KIAA1033*	II	X	
29	*TRAPPC5*	I			65	*UBE2W*	II	X	
30	*EXOC7*	I	X		66	*LOC220930*	II	X	
31	*ACIN1*	I	X		67	*NCRNA00081*	II		X
32	*ITGAL*	I	X		68	*ATP11B*	II	X	
33	*SH2D1B*	I	X		69	*SLC2A1*	II		X
34	*TRMT6*	I	X	X	70	*PIGB*	II		X
35	*NACC1*	I	X		71	*SEPHS2*	II		
36	*RECQL5*	I			72	*FGFR1OP2*	II	X	X

**Table 3:** Twelve candidate genes panel distinguishing steroid sensitive nephrotic syndrome (SSNS) vs. steroid resistant nephrotic syndrome (SRNS) after glucocorticoid (GC) therapy identified with the Integer-Coded Genetic Algorithm (ICGA)—Extreme Learning Machine (ELM) algorithm.

Gene	Protein Function
*RFFL*	Ring Finger and FYVE-Like Domain Containing E3 Ubiquitin Protein Ligase, reported role in endocytosis [37]
*SULF2*	Sulfatase 2, reported role in podocyte physiology and maintenance of glomerular integrity [19,21,27]
*STX4*	Syntaxin 4, reported role in vesicular trafficking [38]
*TRMT6*	tRNA Methyltransferase 6, role in tRNA metabolism [39]
*TOPBP1*	DNA Topoisomerase II Binding Protein 1, reported role in DNA replication [40]
*S1PR5*	Sphingosine-1-Phosphate Receptor 5, reported role in apoptosis, motility and lysosphingolipid signaling [41]
*FAM40A*	Striatin Interacting Protein 1, role in localization of Golgi body and cytoskeletal organization
*KLF2*	Kruppel Like Factor 2, reported role in transcription [42]
*NCRNA00081*	BBSome Interacting Protein 1, reported role in trafficking signal receptors to and from the cilia [43]
*SLC2A1*	Solute Carrier Family 2 Member 1, reported role in glucose transport [44]
*PIGB*	Phosphatidylinositol Glycan Anchor Biosynthesis Class B, reported role in the formation of the glycosylphosphatidyl-inositol anchors of cell surface proteins [45]
*FGFR1OP2*	FGFR1 Oncogene Partner 2, reported role in development of T-cell lymphoma [46]

## Data Availability

The data discussed in this publication have been deposited in NCBI’s Gene Expression Omnibus [30] and are accessible through GEO Series accession number GSE145969 (https://www.ncbi.nlm.nih.gov/geo/query/acc.cgi?acc=GSE145969). The following secure token has been created to allow review of record GSE145969 while it remains in private status: mfexcaqunlmvlwn.

## Supplementary Materials

The following are available online at https://www.mdpi.com/2077-0383/10/3/523/s1, Figure S1: Absence of SULF1 Gene Expression in the Leukocytes of Patients with SSNS and SRNS. Figure S2: VEGF Release from Injured Podocytes.

## Appendix A

### Participating Members of the Pediatric Nephrology Research Consortium (PNRC; Formerly the Midwest Pediatric Nephrology Consortium):

John Mahan and Hiren Patel (Nationwide Children’s Hospital and The Ohio State University, Columbus, OH, USA); Cynthia Pan (Medical College of Wisconsin, Milwaukee, WI, USA); Denis F. Geary (The Hospital for Sick Children, Toronto, ON, Canada); Myra L. Chang (West Virginia University, Charleston, WV, USA); Keisha L. Gibson (University of North Carolina, NC, USA); Franca M. Iorember (Louisiana State University, New Orleans, LA, USA); Patrick D. Brophy (Children’s Hospital, University of Iowa, Iowa City, IA, USA); Tarak Srivastava (Children’s Mercy Hospital, Kansas City, MO, USA); Larry A. Greenbaum (Emory University School of Medicine, Atlanta, GA, USA).

## References

[1] Greenbaum L.A., Benndorf R., Smoyer W.E. (2012). Childhood nephrotic syndrome-current and future therapies. Nat. Rev. Nephrol..

[2] Eddy A.A., Symons J.M. (2003). Nephrotic syndrome in childhood. Lancet.

[3] Nourbakhsh N., Mak R.H. (2017). Steroid-resistant nephrotic syndrome: Past and current perspectives. Pediatr. Health Med..

[4] Cunard R., Kelly C.J. (2002). T cells and minimal change disease. J. Am. Soc. Nephrol..

[5] Wei C., Reiser J. (2011). Minimal change disease as a modifiable podocyte paracrine disorder. Nephrol. Dial. Transplant..

[6] Chiang C.K., Inagi R. (2010). Glomerular diseases: Genetic causes and future therapeutics. Nat. Rev. Nephrol..

[7] Bennett M.R., Piyaphanee N., Czech K., Mitsnefes M., Devarajan P. (2012). NGAL distinguishes steroid sensitivity in idiopathic nephrotic syndrome. Pediatr. Nephrol..

[8] Chuang L.Y., Yang C.H., Li J.C. (2012). A hybrid BPSO-CGA approach for gene selection and classification of microarray data. J. Comput. Biol..

[9] Devignes M.D., Benabderrahmane S., Smail-Tabbone M., Napoli A., Poch O. (2012). Functional classification of genes using semantic distance and fuzzy clustering approach: Evaluation with reference sets and overlap analysis. Int. J. Comput. Biol. Drug Des..

[10] Golub T.R., Slonim D.K., Tamayo P., Huard C., Gaasenbeek M., Mesirov J.P., Coller H., Loh M.L., Downing J.R., Caligiuri M.A. (1999). Molecular classification of cancer: Class discovery and class prediction by gene expression monitoring. Science.

[11] Maji P., Das C. (2012). Relevant and significant supervised gene clusters for microarray cancer classification. IEEE Trans. Nanobiosci..

[12] Peng S., Xu Q., Ling X.B., Peng X., Du W., Chen L. (2003). Molecular classification of cancer types from microarray data using the combination of genetic algorithms and support vector machines. FEBS Lett..

[13] Ramaswamy S., Tamayo P., Rifkin R., Mukherjee S., Yeang C.H., Angelo M., Ladd C., Reich M., Latulippe E., Mesirov J.P. (2001). Multiclass cancer diagnosis using tumor gene expression signatures. Proc. Natl. Acad. Sci. USA.

[14] Saraswathi S., Sundaram S., Sundararajan N., Zimmermann M., Nilsen-Hamilton M. (2011). ICGA-PSO-ELM approach for accurate multiclass cancer classification resulting in reduced gene sets in which genes encoding secreted proteins are highly represented. IEEE/ACM Trans. Comput. Biol. Bioinform..

[15] Tapia E., Ornella L., Bulacio P., Angelone L. (2011). Multiclass classification of microarray data samples with a reduced number of genes. BMC Bioinform..

[16] Wang H., Zhang H., Dai Z., Chen M.S., Yuan Z. (2013). TSG: A new algorithm for binary and multi-class cancer classification and informative genes selection. BMC Med. Genom..

[17] Zhang R., Huang G.B., Sundararajan N., Saratchandran P. (2007). Multi-category classification using an Extreme Learning Machine for microarray gene expression cancer diagnosis. IEEE/ACM Trans. Comput. Biol. Bioinform..

[18] Suresh S., Saraswathi S., Sundararajan N. (2010). Performance enhancement of extreme learning machine for multi-category sparse data classification problems. Eng. Appl. Artif. Intell..

[19] Schumacher V.A., Schlotzer-Schrehardt U., Karumanchi S.A., Shi X., Zaia J., Jeruschke S., Zhang D., Pavenstadt H., Drenckhan A., Amann K. (2011). WT1-dependent sulfatase expression maintains the normal glomerular filtration barrier. J. Am. Soc. Nephrol..

[20] Schenk H., Masseli A., Schroder P., Bolanos-Palmieri P., Beese M., Hegermann J., Brasen J.H., Haller H. (2019). Sulfatases, in Particular Sulf1, Are Important for the Integrity of the Glomerular Filtration Barrier in Zebrafish. Biomed. Res. Int..

[21] Takashima Y., Keino-Masu K., Yashiro H., Hara S., Suzuki T., van Kuppevelt T.H., Masu M., Nagata M. (2016). Heparan sulfate 6-O-endosulfatases, Sulf1 and Sulf2, regulate glomerular integrity by modulating growth factor signaling. Am. J. Physiol. Ren. Physiol..

[22] Morimoto-Tomita M., Uchimura K., Werb Z., Hemmerich S., Rosen S.D. (2002). Cloning and characterization of two extracellular heparin-degrading endosulfatases in mice and humans. J. Biol. Chem..

[23] Nagamine S., Tamba M., Ishimine H., Araki K., Shiomi K., Okada T., Ohto T., Kunita S., Takahashi S., Wismans R.G. (2012). Organ-specific sulfation patterns of heparan sulfate generated by extracellular sulfatases Sulf1 and Sulf2 in mice. J. Biol. Chem..

[24] Rosen S.D., Lemjabbar-Alaoui H. (2010). Sulf-2: An extracellular modulator of cell signaling and a cancer target candidate. Expert Opin. Ther. Targets.

[25] Saad O.M., Ebel H., Uchimura K., Rosen S.D., Bertozzi C.R., Leary J.A. (2005). Compositional profiling of heparin/heparan sulfate using mass spectrometry: Assay for specificity of a novel extracellular human endosulfatase. Glycobiology.

[26] Viviano B.L., Paine-Saunders S., Gasiunas N., Gallagher J., Saunders S. (2004). Domain-specific modification of heparan sulfate by Qsulf1 modulates the binding of the bone morphogenetic protein antagonist Noggin. J. Biol. Chem..

[27] Eremina V., Jefferson J.A., Kowalewska J., Hochster H., Haas M., Weisstuch J., Richardson C., Kopp J.B., Kabir M.G., Backx P.H. (2008). VEGF inhibition and renal thrombotic microangiopathy. N. Engl. J. Med..

[28] Sison K., Eremina V., Baelde H., Min W., Hirashima M., Fantus I.G., Quaggin S.E. (2010). Glomerular structure and function require paracrine, not autocrine, VEGF-VEGFR-2 signaling. J. Am. Soc. Nephrol..

[29] Hart S.N., Therneau T.M., Zhang Y., Poland G.A., Kocher J.P. (2013). Calculating sample size estimates for RNA sequencing data. J. Comput. Biol..

[30] Edgar R., Domrachev M., Lash A.E. (2002). Gene Expression Omnibus: NCBI gene expression and hybridization array data repository. Nucleic Acids Res..

[31] Goldberg D. (1989). Genetic Algorithms in Search, Optimization and Machine Learning.

[32] Mitchell M. (1998). An Introduction to Genetic Algorithms.

[33] Huang G.B., Zhu Q.Y., Siew C.K. (2006). Extreme learning machine: Theory and application. Neurocomputing.

[34] Saraswathi S., Fernandez-Martinez J.L., Kolinski A., Jernigan R.L., Kloczkowski A. (2012). Fast learning optimized prediction methodology (FLOPRED) for protein secondary structure prediction. J. Mol. Model..

[35] Uchimura K., Morimoto-Tomita M., Rosen S.D. (2006). Measuring the activities of the Sulfs: Two novel heparin/heparan sulfate endosulfatases. Methods Enzymol..

[36] Agrawal S., Chanley M.A., Westbrook D., Nie X., Kitao T., Guess A.J., Benndorf R., Hidalgo G., Smoyer W.E. (2016). Pioglitazone Enhances the Beneficial Effects of Glucocorticoids in Experimental Nephrotic Syndrome. Sci. Rep..

[37] Coumailleau F., Das V., Alcover A., Raposo G., Vandormael-Pournin S., Le Bras S., Baldacci P., Dautry-Varsat A., Babinet C., Cohen-Tannoudji M. (2004). Over-expression of Rififylin, a new RING finger and FYVE-like do-main-containing protein, inhibits recycling from the endocytic recycling compartment. Mol. Biol. Cell.

[38] Mandon B., Chou C.L., Nielsen S., Knepper M.A. (1996). Syntaxin-4 is localized to the apical plasma membrane of rat renal collecting duct cells: Possible role in aquaporin-2 trafficking. J. Clin. Investig..

[39] Ozanick S., Krecic A., Andersland J., Anderson J.T. (2005). The bipartite structure of the tRNA m1A58 methyltransferase from S. cerevisiae is conserved in humans. RNA.

[40] Kumagai A., Shevchenko A., Dunphy W.G. (2010). Treslin collaborates with TopBP1 in triggering the initiation of DNA rep-lication. Cell.

[41] Im D.S., Heise C.E., Ancellin N., O’Dowd B.F., Shei G.J., Heavens R.P., Rigby M.R., Hla T., Mandala S., McAllister G. (2000). Characterization of a novel sphingosine 1-phosphate receptor, Edg-8. J. Biol. Chem..

[42] Richardson M.W., Jadlowsky J., Didigu C.A., Doms R.W., Riley J.L. (2012). Kruppel-like factor 2 modulates CCR5 expression and susceptibility to HIV-1 infection. J. Immunol..

[43] Loktev A.V., Zhang Q., Beck J.S., Searby C.C., Scheetz T.E., Bazan J.F., Slusarski D.C., Sheffield V.C., Jackson P.K., Nachury M.V. (2008). A BBSome subunit links ciliogenesis, microtubule stability, and acetylation. Dev. Cell.

[44] Baroni M.G., Oelbaum R.S., Pozzilli P., Stocks J., Li S.R., Fiore V., Galton D.J. (1992). Polymorphisms at the GLUT1 (HepG2) and GLUT4 (muscle/adipocyte) glucose transporter genes and non-insulin-dependent diabetes mellitus (NIDDM). Hum. Genet..

[45] Takahashi M., Inoue N., Ohishi K., Maeda Y., Nakamura N., Endo Y., Fujita T., Takeda J., Kinoshita T. (1996). PIG-B, a membrane protein of the endoplasmic reticulum with a large lumen-al domain, is involved in transferring the third mannose of the GPI anchor. EMBO J..

[46] Grand E.K., Grand F.H., Chase A.J., Ross F.M., Corcoran M.M., Oscier D.G., Cross N.C. (2004). Identification of a novel gene, FGFR1OP2, fused to FGFR1 in 8p11 myelopro-liferative syndrome. Genes Chromosom. Cancer.

[47] Okamoto K., Tokunaga K., Doi K., Fujita T., Suzuki H., Katoh T., Watanabe T., Nishida N., Mabuchi A., Takahashi A. (2011). Common variation in GPC5 is associated with acquired nephrotic syndrome. Nat. Genet..

[48] Singer M.S., Phillips J.J., Lemjabbar-Alaoui H., Wang Y.Q., Wu J., Goldman R., Rosen S.D. (2015). SULF2, a heparan sulfate endosulfatase, is present in the blood of healthy individuals and increases in cirrhosis. Clin. Chim. Acta.

[49] Tang R., Rosen S.D. (2009). Functional consequences of the subdomain organization of the sulfs. J. Biol. Chem..

[50] Ai X., Do A.T., Kusche-Gullberg M., Lindahl U., Lu K., Emerson C.P. (2006). Substrate specificity and domain functions of extracellular heparan sulfate 6-O-endosulfatases, QSulf1 and QSulf2. J. Biol. Chem..

[51] Diez-Roux G., Ballabio A. (2005). Sulfatases and human disease. Annu. Rev. Genom. Hum. Genet..

[52] Sardiello M., Annunziata I., Roma G., Ballabio A. (2005). Sulfatases and sulfatase modifying factors: An exclusive and promiscuous relationship. Hum. Mol. Genet..

[53] Iozzo R.V. (2005). Basement membrane proteoglycans: From cellar to ceiling. Nat. Rev. Mol. Cell Biol..

[54] Kolset S.O., Reinholt F.P., Jenssen T. (2012). Diabetic nephropathy and extracellular matrix. J. Histochem. Cytochem. Off. J. Histochem. Soc..

[55] Khalil R., Lalai R.A., Wiweger M.I., Avramut C.M., Koster A.J., Spaink H.P., Bruijn J.A., Hogendoorn P.C.W., Baelde H.J. (2019). Glomerular permeability is not affected by heparan sulfate glycosaminoglycan deficiency in zebrafish embryos. Am. J. Physiol. Ren. Physiol..

[56] Eremina V., Quaggin S.E. (2004). The role of VEGF-A in glomerular development and function. Curr. Opin. Nephrol. Hypertens..

[57] Eremina V., Baelde H.J., Quaggin S.E. (2007). Role of the VEGF—A signaling pathway in the glomerulus: Evidence for crosstalk between components of the glomerular filtration barrier. Nephron Physiol..

[58] Schrijvers B.F., Flyvbjerg A., De Vriese A.S. (2004). The role of vascular endothelial growth factor (VEGF) in renal pathophysiology. Kidney Int..

[59] Estrada C.C., Maldonado A., Mallipattu S.K. (2019). Therapeutic Inhibition of VEGF Signaling and Associated Nephrotoxicities. J. Am. Soc. Nephrol..

[60] Nagarajan A., Malvi P., Wajapeyee N. (2018). Heparan Sulfate and Heparan Sulfate Proteoglycans in Cancer Initiation and Progression. Front. Endocrinol..

[61] Agrawal S., Merchant M.L., Kino J., Li M., Wilkey D.W., Gaweda A.E., Brier M.E., Chanley M.A., Gooding J.R., Sumner S.J. (2020). Predicting and Defining Steroid Resistance in Pediatric Nephrotic Syndrome Using Plasma Proteomics. Kidney Int. Rep..

[62] Gooding J.R., Agrawal S., McRitchie S., Acuff Z., Merchant M.L., Klein J.B., Smoyer W.E., Sumner S.J., Midwest Pediatric Nephrology C. (2020). Predicting and Defining Steroid Resistance in Pediatric Nephrotic Syndrome Using Plasma Metabolomics. Kidney Int. Rep..

[63] Agrawal S., Brier M.E., Kerlin B.A., Smoyer W.E., Consortium P.N.R. (2021). Plasma Cytokine Profiling to Predict Steroid Resistance in Nephrotic Syndrome. Kidney Int. Rep..

