# Supplementary material for: Sulfatase 2 Is Associated with Steroid Resistance in Childhood Nephrotic Syndrome
Source: J Clin Med. 2021 Feb 2;10(3):523. doi: 10.3390/jcm10030523 (PMC7867139; doi:10.3390/jcm10030523)
Supplement: Supplementary file 1 [file jcm-10-00523-s001.pdf]

Supplementary Material: SULF2 in SRNS

Materials and Methods

Pediatric Nephrotic Syndrome Patients and Sample Collection

Clinical data were collected from all patients at the time of enrollment (Pre-treatment sample) and at the time of the second visit (Post-treatment sample). **Table 1** describes the demographics of the patients included in this study. Paired plasma samples were collected for each patient, with the Pre-treatment sample collected at the time of disease presentation, ideally before even a single dose of GC, and the second sample Post-treatment sample after ~7 weeks of GC therapy when the clinical determination of SSNS versus SRNS had been determined by the treating nephrologist.

Pre-treatment samples from 68% of the patients were GC naïve, and none of the patients received >8 days of GC therapy prior to the Pre-treatment sample collection. Each blood sample consisted of: a) 8 ml of peripheral blood drawn into a CPT® tube with sodium citrate anticoagulant (Becton & Dickenson, Franklin Lakes, NJ), and b) 2.5 ml of peripheral blood drawn into a PaxGene® tube (PreAnalytiX GmbH, Switzerland). Samples in CPT tubes were processed immediately to isolate peripheral blood leukocytes and plasma, or, depending on the participating center, were shipped overnight at ambient temperature before processing. Leukocytes were isolated from blood by standard protocols and stored at -80°C. Frozen PaxGene tubes were processed to isolate total peripheral blood RNA according to the manufacturer's instructions (PreAnalytiX).

Paired samples (Pre, Post) from 12 patients (8 SSNS, 4SRNS) were processed for RNAseq analysis. Samples from 42 patients (28 SSNS, 14 SRNS) were processed for qRT-PCR gene expression validation analysis, 45 patients (30 SSNS, 15 SRNS) for arylsulfatase activity assays, 37 patients (25 SSNS, 13 SRNS) for endosulfatase activity or VEGF binding assays, and 40 patients (27 SSNS, 13 SRNS) for plasma VEGF quantitation by ELISA (see below).

Sequencing, Analyses and Data Access

Total leukocyte RNA was extracted using Trizol reagent (Life Technologies, Grand Island, NY) according to the manufacturer's instructions. RNA quality was verified on a 2100 Bioanalyzer (Agilent, Santa Clara, CA), with all samples showing an RNA integrity number >6.5. NuGen Ovation RNASEQ System (San Carlos, CA) was used to generate cDNA libraries from 10 ng of total RNA per sample (NuGen Technologies, San Carlos, CA). This kit employs poly-dT and random hexamer primers for cDNA synthesis, the latter designed to reduce cDNAs from rRNA by at least 90%. This NuGen library preparation is able to capture RNA lacking polyA tails, including ncRNA RNAs, although in this study we focused sequence analysis on annotated coding genes[1]. A linear amplification step enables RNAseq analysis of small amounts of RNA (we used 10-20 ng total RNA). Samples were processed to produce 8-barcoded, paired-end libraries or 16-barcoded, single-end libraries. Samples were sequenced using the SOLiD 5500 Next-Generation Sequencing instrument (Life Technologies).

RNAseq data from 8 SSNS and 4 SRNS patients (paired Pre- and Post-treatment samples, from before and after GC treatment, respectively) were collected, and the number of sequence fragments generated and mapping percentages had an average of ~60% of generated reads for each sample mapping to the genome (not shown). Overall, 5-112 million fragments were generated per sample. One SSNS patient sample gave poor reads and was thus excluded from further analysis. RNAseq sequences were aligned to the International Human Genome Sequencing Consortium human reference sequence (hg19 refGene annotation from the University of California at Santa Cruz genome browser) containing IUPAC codes for known dnSNP variants using SOLiDLifescope v2.5.1 (Life Technologies). Although read alignments were concentrated into peaks at known exons, other regions of the genome such as introns and intergenic spaces also showed read coverage, which were excluded from this study [1]. For samples included in an 8-barcode set, paired-end reads were aligned (with 50 bases sequenced in the forward direction and 35 bases for the reverse), whereas for

samples included in a 16-barcode set, single-end alignment was performed for the 75 bases sequenced in the forward direction. Gene expression was measured in reads per kilobase per million (RPKM), as reported by the wtcounts module of Lifescope calculated based on reads that aligned to coding regions of genes annotated by RefSeq [1][2, 3].

The primary data set consisted of expression values for >20,000 genes, which were reduced to 7,334 genes by the removal of those genes with little or no expression in one or several samples. These expression values were used to calculate the gene expression ratios (GER) before (Pre) and after (Post) treatments with GC (GER[Pre/Post]).

Selection of Candidate Genes Using Statistical Analyses

To identify a smaller set of genes that could discriminate between SSNS vs. SRNS patient groups, "volcano plotting" was initially employed. This representation combines a statistical test with the magnitude of the change in gene expression induced by the treatment, enabling identification of responsive genes that potentially discriminate between the SSNS and SRNS patient groups[4]. For each gene, the difference in the gene expression ratios (GER; Pre/Post) of all samples in the SSNS patient group (r1) and in the SRNS patient group (r2) was formed. This difference (r1-r2) was plotted on the abscissa against $-\log_{10}$(p-value) on the ordinate. To obtain the *P*-values, a two-sample unpaired *t*-test with permutations of the sample labels (1000 permutations) was used. Two cutoff values were applied: C_1= ($\log_2$(1.2)= 0.263 for the mean ratio difference, and C_2 = 0.02 for the *P*-values. Genes that met the conditions $|r1-r2| > C_1$ and p-value $< C_2$ were selected.

Expression pattern relationships among these candidate genes (72) were determined using a hierarchical clustering method which calculates the pairwise (Euclidian) distances between all pairs of genes[5, 6]. In addition, scatter plotting was applied as a statistical tool in order to separate averaged GER (Pre/Post) values for both groups of patients with SRNS and SSNS. In these plots, "linear separation" was defined as the ability to separate two groups of data points by a straight line.

Selection of Candidate Genes Using the ICGA-ELM Algorithm

Following the statistical analyses, the Integer-Coded Genetic Algorithm (IBCGA) based on the GA[7-9] and the neural network-based Extreme Learning Machine (ELM) algorithm [9-11], jointly known as the ICGA-ELM algorithm, were used to obtain a small set of genes that reliably discriminated between the SSNS and SRNS patient groups. The GA mimics adaptive natural processes and has been widely applied to reduce features, using a wrapper method [9, 12]. In our study, the GA was used to identify the smallest set of genes (out of the 72 genes) that could discriminate between the SRNS and SSNS patients with high accuracy. The ICGA produces the solution as a string of integer codes representing the inclusion or exclusion of genes [7-9]. Various combinations of genes are evaluated to determine if they are able to classify the data (e.g. the patient groups) with high accuracy (fitness criteria). The representative string evolves during the iterative model building process through the application of different genetic operators such as inheritance, mutation, selection, and crossover. The fitness function (highest accuracy of classification) is evaluated by the ELM algorithm with the current set of genes during the iterations. The process of selecting an improved set of genes during successive generations continues until the termination (convergence) criterion is satisfied. Once this procedure is finished, post processing is performed to analyze the relative frequency of the genes that appear most frequently in the successful classification.

The ELM algorithm is a feed-forward neural network that helps to evaluate the identified genes. Neural networks have been widely used for pattern recognition and feature extraction on numerous applications in bioinformatics [9, 11, 13, 14]. Neural networks are capable of discerning associations and searching for regularities within a set of patterns that are present in the input samples. A set of parameters that represent these associations and patterns are stored during model development and are subsequently used to correctly determine the class to which new inputs belong.

In our study, during a single ICGA-ELM iteration, the algorithm searched for a set of optimal parameters which, in combination with the input expression values, determined whether the patient belonged to the SSNS or the SRNS group. The genes identified by the ICGA-ELM algorithm are thus potential biomarkers to distinguish patients with SRNS from those with SSNS.

Gene Expression Validation by qRT-PCR

Peripheral blood cDNA was produced from patient whole-blood RNA (BioChain, Hayward, CA) by reverse transcriptase (High Capacity kit, Life Technologies). qRT-PCR was performed on a iQ5 Real-Time PCR Detection System (Bio-Rad, Hercules, CA) using SYBR Green master mix (BioRad). The cycle parameters were 5 min at 95°C, 40 cycles of 15 s at 95°C, and 30 s at 55°C. Melting curves were obtained to ensure single products. Expression was measured by the Pfaffl method [15], with expression of the housekeeping gene *RPL19* (encoding the ribosomal protein L19), which is known to not be affected by GC [16, 17], used for normalization. The following primer sequences were used: tggaacctgggatctttctg and gctgattcaaaatgcctcgt for *SULF1*, acacgtactggtgcatgagg and gcttgtaacccttgcagctc for *SULF2*, and tgtacctgaaggtcaaagggaatgtg and ttcttggtctcctcctccttggac for *RPL19*. Commercially available RNA from human kidneys (BioChain, Newark, CA) was used as control template for *SULF1* to demonstrate the proper functioning of the primers.

Arylsulfatase Activity of SULF2 in the Plasma of Nephrotic Syndrome Patients

Arylsulfatase activity of SULF2 was measured in the NS patient plasma samples using a slight modification of a previously described assay [18]. Briefly, plasma was clarified by centrifugation at 15,000 x g for 30 min at 4°C, and 10 µl of supernatant (excluding any of the upper, lipid-rich layer) was combined with stock solutions to a final concentration of 50 mM HEPES-KOH, pH 8.0, 10 mM lead acetate, and 10 mM 4-methylumbelliferyl sulfate in a total volume of 50 µl.

The enzymatic activity of SULF2 (and of SULF1) can be measured at pH 8.0, whereas the activities of all other sulfatases are suppressed at this pH. After incubation at 37°C for 2 h, 40 µl of this mixture was combined with 200 µl of 0.5 M Na_2CO_3/$NaHCO_3$, pH 10.7, and fluorescence was measured in black-walled clear bottom plates at 460 nm emission with excitation at 360 nm. Heat-inactivated plasma for 30 min at 95°C was used as a negative control.

VEGF Binding Assay in the Plasma of Nephrotic Syndrome Patients

The endosulfatase activity of SULF2 in patient plasma was measured by studying its effect on the binding of VEGF to immobilized heparin in an ELISA assay, as previously reported [18]. Briefly, wells of a 96-well plate (Immulon 2HB) were coated with 100 ul of 100ng/ml of heparin-BSA in PBS at 4°C overnight. After washing 3X with PBS-T (Tween-20 at 0.1%), wells were blocked with 100 ul of blocking reagent (3% BSA, 0.01% NaN_3 in PBS) for 2 h at RT. Plates were washed 3X with PBS-T and 100 ul of sample added in triplicates and incubated overnight at 37C. The 100 ul sample contained 10 ul of patient plasma in 5 umol of HEPES, pH 7.6 and 1umol of MgCl2 in ddH2O. Wells were washed 3X with PBS-T and incubated with 25 ul of 25 nM $VEGF_{165}$ at RT for 30 minutes. Wells were washed 3X with PBS-T and incubated with 100 ul of 1ug/ml goat anti-human VEGF antibody in 0.1%BSA in PBS at RT for 1 h. Wells were washed 3X with PBS-T, incubated with 1.2 ug/ml secondary anti-goat antibody for 30 minutes at RT, washed 3X with PBS-T and incubated with 2 ug/ml of an alkali-phosphatase conjugated streptavidin for 30 minutes at RT. Wells were washed again 3X with PBS-T and incubated with 100 ul of PNPP in diethanolamine buffer, pH 9.8 at RT for 5 minutes. Absorbance was measured at 405 nm and the OD values subtracted from negative control (no VEGF added and H2O as sample), divided by the positive control value (VEGF added and H2O as sample, representing 100% VEGF binding after subtracting from the negative control reading) and graphed as %VEGF bound on the y axis. Any negative values were plotted as 1 for ratio calculations.

VEGF Quantitation in the Plasma of Nephrotic Syndrome Patients

The total amount of VEGF in the Pre- and Post-treatment plasma samples from 40 patients (27 SSNS and 13 SRNS) was quantified using a commercial Human VEGF Quantikine ELISA kit assay (R&D Systems Inc., Minneapolis, MN) per the manufacturer's instructions. A standard curve for VEGF concentrations ranging from 8-6,000 pg/ml vs. OD was generated with recombinant VEGF, and the ideal patient sample volume determined based on preliminary assays. The final readings were averaged and extrapolated on the standard curve and plotted using Graphpad software. Readings that were Out of Range were plotted as 1 for ratio calculations.

Animal Nephropathy Model

Animal studies were approved by the Institutional Animal Care and Use Committee at Nationwide Children's Hospital and the experiments were performed in accordance with the approved guidelines. Proteinuria was induced in male Wistar rats weighing ~150–200 g by a single intravenous (IV) PAN (Sigma-Aldrich, St. Louis, MO) injection (50 mg/kg) on Day 0, while the control group received IV saline injections (n = 3 per group). A larger group of these studies have been previously described and reported [19]. Urine samples were collected at baseline (prior to injury) and daily during the study for analysis and urine protein:creatinine ratios (UPC) were measured by Antech Diagnostics GLP (Morrisville, NC), which is fully compliant with Good Laboratory Practice (GLP) regulations. The rats were weighed daily and sacrificed on Day 11 at the point of expected peak proteinuria and the kidneys harvested, weighed, and processed for glomerular isolation. Glomeruli were isolated using a standard sequential sieving method, as we have described previously [19].

Podocyte Culture and Treatments, SULF2 mRNA Expression and VEGF Quantitation

The conditionally immortalized human podocyte cell line (kind gift from Moin Saleem) was cultured as previously described [20]. Differentiated podocytes were injured with 100 ug/ml PAN (Sigma-Aldrich) for short-term injury experiments, and 5 ug/ml PAN for long-term injury experiments. Additionally, a subset of PAN-treated podocytes was treated with glucocorticoids (1 uM dexamethasone; Sigma-Aldrich) and cells were harvested at 6 h and 24 h (representing short-term injury), and 7 d and 10 d (representing long-term injury). VEGF quantitation in the supernatant of treated podocytes was performed as described above.

RNA Isolation and RT-PCR

Total RNA was isolated from rat glomeruli and human cultured podocytes using the RNeasy kit (Qiagen) according to the manufacturer's instructions. The purity and yield of RNA was determined by measuring the absorbance at 260 and 280 nm and used for quantitative reverse transcribed-polymerase chain reaction (qRT-PCR), as described previously [19]. Briefly, 1 µg of RNA was digested with DNase (Ambion, Thermo Fisher Scientific) at 37 °C for 30 min to eliminate any genomic DNA, followed by DNase inactivation with 5 mM EDTA at 75 °C for 10 min. 1 µg DNase-treated RNA was reverse-transcribed to cDNA in a 20 µl reaction using iScript reverse transcriptase (Bio-Rad, Hercules, CA), according to manufacturer's protocol. The *SULF2*, *GAPDH* and *RPL19* mRNAs were quantified by qRT-PCR using the SYBR green method on an iQ5 thermal cycler (Bio-Rad) using forward and reverse primers. The following primer sequences were used in addition to the human primer sequences described above: atgcatcccagcacatcaca and tgaatgggcttcatgggtcc for rat *SULF2*, aaacccatcaccatcttcca and gtggttcacacccatcacaa for rat GAPDH. The PCR conditions were as follows: 1 cycle at 95 °C for 3 min, 40 cycles at 95 °C for 10 sec, and 55 °C for 10 sec, followed by a melt curve analysis. The amplification efficiency of each primer pair was measured, and values were normalized to the housekeeping gene *GAPDH* or *RPL19* and plotted as fold changes relative to control treatments.

Results

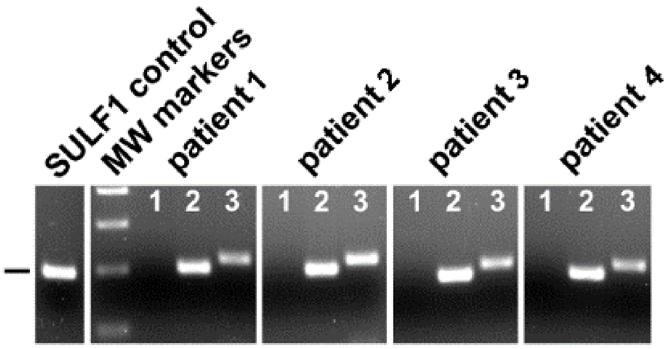

Supplementary Figure S1. Absence of *SULF1* Gene Expression in the Leukocytes of Patients with SSNS and SRNS. cDNA was synthesized and used for determination of *SULF1* and *SULF2* expression. The relative expression of *SULF1* and *SULF2* was then determined by RT-PCR. 35 cycles of RT-PCR failed to amplify *SULF1* (lanes labeled with "1") in peripheral blood leukocyte samples, in contrast to *SULF2* (lanes labeled with "2") and *RPL19* (lanes labeled with "3"). Results from 4 representative patient samples are shown. As a positive control reaction to the *SULF1* primers, RT-PCR was also performed using cDNA from whole human kidneys as the template (*SULF1* control). The primer design resulted in amplicons of 196 bp (*SULF1*), 198 bp (*SULF2*), and 220 bp (*RPL19*). The position of the 200 bp molecular mass marker band is indicated by the bar on the left.

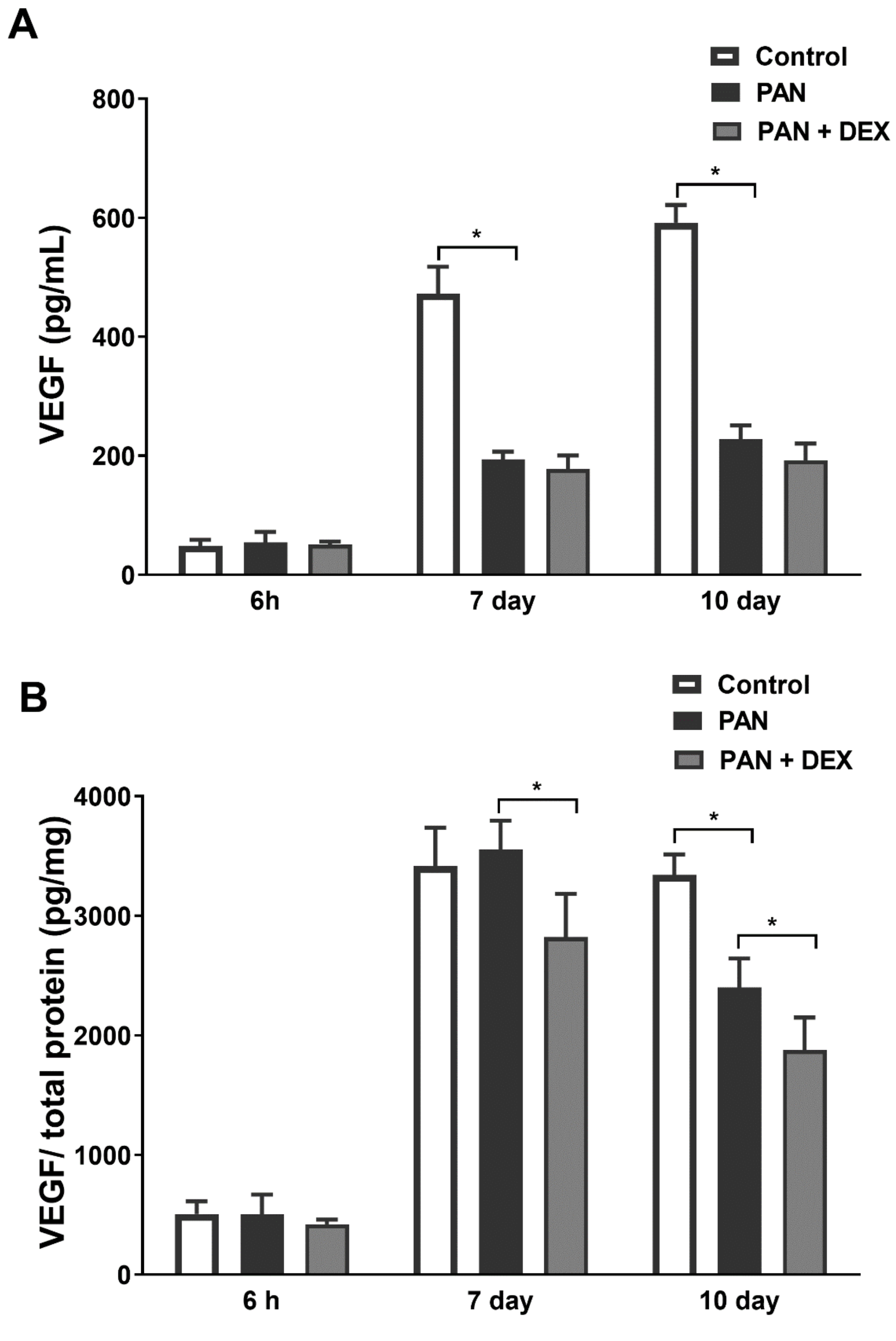

Supplementary Figure S2. Time Course of VEGF-A Release from Human Podocytes after PAN Injury with and without Glucocorticoid Exposure. **A)** Total VEGF release in the culture media. **B)** VEGF release normalized to total cellular protein content.

References

1. Webb A, Papp AC, Sanford JC et al. Expression of mRNA transcripts encoding membrane transporters detected with whole transcriptome sequencing of human brain and liver. Pharmacogenet Genomics 2013; 23: 269-278.
2. Meyer LR, Zweig AS, Hinrichs AS et al. The UCSC Genome Browser database: extensions and updates 2013. Nucleic Acids Res 2013; 41: D64-69.
3. Pruitt KD, Tatusova T, Maglott DR. NCBI Reference Sequence (RefSeq): a curated non-redundant sequence database of genomes, transcripts and proteins. Nucleic Acids Res 2005; 33: D501-504.
4. Cui X, Churchill GA. Statistical tests for differential expression in cDNA microarray experiments. Genome Biol 2003; 4: 210.
5. Szekely GJ, Rizzo ML. Hierarchical clustering via Joint Between-Within Distances: Extending Ward's Minimum Variance Method. Journal of Classification 2005; 22: 151-183.
6. Ward JH. Hierarchical Grouping to Optimize an Objective Function. Journal of the American Statistical Association 1963; 58: 236-244.
7. Goldberg D. Genetic Algorithms in Search, Optimization and Machine Learning: Addison Wesley. New York, NY,1989.
8. M M. An Introduction to genetic algorithms. Cambridge, Massachuesetts: First MIT Press,1998.
9. Suresh S, Saraswathi S, Sundararajan N. Performance enhancement of extreme learning machine for multi-category sparse data classification problems. Engineering Applications of Artificial Intelligence 2010; 23: 1149-1157.
10. Huang GB, Zhu QY, Siew CK. Extreme learning machine: Theory and application. Neurocomputing 2006; 70: 489-501.
11. Saraswathi S, Fernandez-Martinez JL, Kolinski A et al. Fast learning optimized prediction methodology (FLOPRED) for protein secondary structure prediction. J Mol Model 2012; 18: 4275-4289.
12. Holland JH. Generic Algorithms. Scientific American 1992; 267: 44-50.
13. Ramaswamy S, Tamayo P, Rifkin R et al. Multiclass cancer diagnosis using tumor gene expression signatures. Proc Natl Acad Sci U S A 2001; 98: 15149-15154.
14. Saraswathi S, Sundaram S, Sundararajan N et al. ICGA-PSO-ELM approach for accurate multiclass cancer classification resulting in reduced gene sets in which genes encoding secreted proteins are highly represented. IEEE/ACM Trans Comput Biol Bioinform 2011; 8: 452-463.
15. Pfaffl MW, Lange IG, Daxenberger A, Meyer HH. Tissue-specific expression pattern of estrogen receptors (ER): quantification of ER alpha and ER beta mRNA with real-time RT-PCR. APMIS 2001; 109: 345-355.
16. Chen W, Dang T, Blind RD et al. Glucocorticoid receptor phosphorylation differentially affects target gene expression. Mol Endocrinol 2008; 22: 1754-1766.
17. Holmstrom SR, Chupreta S, So AY, Iniguez-Lluhi JA. SUMO-mediated inhibition of glucocorticoid receptor synergistic activity depends on stable assembly at the promoter but not on DAXX. Mol Endocrinol 2008; 22: 2061-2075.
18. Uchimura K, Morimoto-Tomita M, Rosen SD. Measuring the activities of the Sulfs: two novel heparin/heparan sulfate endosulfatases. Methods Enzymol 2006; 416: 243-253.
19. Agrawal S, Chanley MA, Westbrook D et al. Pioglitazone Enhances the Beneficial Effects of Glucocorticoids in Experimental Nephrotic Syndrome. Sci Rep 2016; 6: 24392.
20. Agrawal S, Guess AJ, Chanley MA, Smoyer WE. Albumin-induced podocyte injury and protection are associated with regulation of COX-2. Kidney Int 2014; 86: 1150-1160.
